# Plant versus pollinator protection: balancing pest management against floral contamination for insecticide use in Midwestern US cucurbits

**DOI:** 10.1093/jee/toae202

**Published:** 2024-09-15

**Authors:** Keng-Lou James Hung, John J Ternest, Thomas J Wood, Laura L Ingwell, Elias H Bloom, Zsofia Szendrei, Ian Kaplan, Karen Goodell

**Affiliations:** Department of Evolution, Ecology and Organismal Biology, The Ohio State University, Newark, OH, USA; Oklahoma Biological Survey, School of Biological Sciences, University of Oklahoma, Norman, OK, USA; Department of Entomology, Purdue University, West Lafayette, IN, USA; Department of Entomology and Nematology, University of Florida, Gainesville, FL, USA; Department of Entomology, Michigan State University, East Lansing, MI, USA; NL Biodiversity and Society, Naturalis Biodiversity Center, Leiden, the Netherlands; Department of Entomology, Purdue University, West Lafayette, IN, USA; Department of Entomology, Michigan State University, East Lansing, MI, USA; Department of Plant Pathology and Plant-Microbe Biology, School of Integrative Plant Science, Cornell University, Ithaca, NY, USA; Department of Entomology, Michigan State University, East Lansing, MI, USA; Department of Entomology, Purdue University, West Lafayette, IN, USA; Department of Evolution, Ecology and Organismal Biology, The Ohio State University, Newark, OH, USA

**Keywords:** cucumber beetle, insecticide residue, neonicotinoid, pollen hazard quotient, window of protection

## Abstract

Controlling crop pests while conserving pollinators is challenging, particularly when prophylactically applying broad-spectrum, systemic insecticides such as neonicotinoids. Systemic insecticides are often used in conventional agriculture in commercial settings, but the conditions that optimally balance pest management and pollination are poorly understood. We investigated how insecticide application strategies control pests and expose pollinators to insecticides with an observational study of cucurbit crops in the Midwestern United States. To define the window of protection and potential pollinator exposure resulting from alternative insecticide application strategies, we surveyed 62 farms cultivating cucumber, watermelon, or pumpkin across 2 yr. We evaluated insecticide regimes, abundance of striped and spotted cucumber beetles (*Acalymma vittatum* [Fabricius] and *Diabrotica undecimpunctata* Mannerheim), and insecticide residues in leaves, pollen, and nectar. We found that growers used neonicotinoids (thiamethoxam and imidacloprid) at planting in all cucumber and pumpkin and approximately half of watermelon farms. In cucumber, foliar thiamethoxam levels were orders of magnitude higher than the other crops, excluding nearly all beetles from fields. In watermelon and pumpkin, neonicotinoids applied at planting resulted in 4–8 wk of protection before beetle populations increased. Floral insecticide concentrations correlated strongly with foliar concentrations across all crops, resulting in high potential exposure to pollinators in cucumber and low-moderate exposure in pumpkin and watermelon. Thus, the highest-input insecticide regimes maintained cucumber beetles far below economic thresholds while also exposing pollinators to the highest pollen and nectar insecticide concentrations. In cucurbits, reducing pesticide inputs will likely better balance crop protection and pollination, reduce costs, and improve yields.

## Introduction

Since the 1940s, global agriculture has increasingly relied on insecticides to enhance crop productivity by mitigating pest damage (Tilman et al. 2002, Carvalho 2017). While yields in most major crops have increased due to agricultural intensification over this period, these yield increases come with risks. To derive the greatest benefit of insecticide application, growers must balance positive effects of pest control with negative effects on beneficial organisms such as pollinators (Egan et al. 2020). This balance is determined by the relative importance of pest versus beneficial arthropods, which vary across crops. We expect that pollinator-dependent crops will have a lower net return when growers apply insecticides, due to non-target effects on bees and other pollinators, which can compromise fruit production (Stanley et al. 2015, Pecenka et al. 2021). The balance between positive and negative outcomes also depends on features inherent to the insecticide used, including exposure route (e.g., contact vs. systemic), residual activity, and selectivity for target pests. The decisions of growers, including frequency of insecticide use and the correspondence between initiating control measures and pest damage (e.g., prophylactic vs. reactionary approaches), also influence the balance between positive and negative outcomes (Ternest et al. 2020). Broad-spectrum, systemic insecticides that are applied prophylactically when pest populations are below economic thresholds are more likely to result in a net negative effect (Douglas et al. 2015). However, few studies have quantified and compared crop protection benefits against non-target impacts to estimate the overall value of insecticides (but see Pecenka et al. 2021).

Pollinators are highly vulnerable to systemic insecticides (Chagnon et al. 2015), which are frequently applied as seed coatings or soil drenches to reduce the need for spraying foliar insecticides that can drift onto neighboring plants and soil. Neonicotinoids are systemic insecticides that often effectively control insect pests, yet neonicotinoids can also translocate into pollen and nectar at high concentrations, resulting in exposure to pollinators (Goulson 2013, Zioga et al. 2020) and corresponding negative impacts from sublethal and behavioral effects to population-level effects over multiple generations (Stuligross and Williams 2021, Willis Chan and Raine 2021, Herbertsson et al. 2022).

To estimate the overall value of systemically applied neonicotinoids in pollinator-dependent crops, it is critical to gauge their potential benefits in pest management by quantifying their “window of protection”—that is, how long they shield seedlings from pests early in the season when seedlings are most vulnerable to damage. It is also critical to consider those benefits in light of potential risks by quantifying the hazard posed to pollinators through exposure to the insecticides via pollen and nectar in flowers that appear later in the growing season. This dual pest-pollinator framework is an important consideration for balancing the benefits and costs of systemic insecticide application, but detailed knowledge is absent for many crops where neonicotinoids are used. We can point to very few systems where the duration of early-season protection in foliage and non-target exposure in later-season flowers are measured in tandem (Pecenka et al. 2021).

Cucurbits are important vegetable crops attacked by insect pests that can reduce yield by defoliating seedlings and transmitting lethal pathogens. The spotted cucumber beetle (*Diabrotica undecimpunctata* Mannerheim) and striped cucumber beetle (*Acalymma vittatum* [Fabricius]) comprise the most significant insect pests of cucurbit crops in North America (Foster 2016, Haber et al. 2021). These beetles act as vectors for pathogens such as bacterial wilt (*Erwinia tracheiphila* [Smith]) (Rojas et al. 2015), which is an economically important cucurbit disease. The economic injury level of cucumber beetles ranges from 1 to 5 beetles per plant, depending on the cucurbit variety, growth stage, and susceptibility to disease (Brust and Foster 1999, Foster 2016), but growers typically apply neonicotinoids prophylactically at planting as a seed treatment or soil drench, independent of beetle presence or density. Additionally, since protection by neonicotinoids wanes over time, many growers apply subsequent foliar insecticide sprays, with applications beginning as soon as 2 wk after planting (Ternest et al. 2020). Insecticide effects on non-target insects may be especially consequential for cucurbit crops, as their monoecious mating system requires insect pollinators to transfer pollen from male to female flowers. Recent work in watermelon (Pecenka et al. 2021) and squash (Obregon et al. 2022) indicates that neonicotinoid use at planting can be detrimental for pollinators, despite its pest management benefits. On-farm studies can help strengthen the inferences from these experimental studies that simulate grower pest management by capturing the dynamic conditions of real-world agricultural practices that cause growers to adjust their application rates, timing, and products.

Here, we examined 3 cucurbit crops—pickling cucumber, pumpkin, and watermelon—in the Midwestern United States to understand how insecticide use simultaneously shapes insect pest pressure by striped and spotted cucumber beetles and potential harm to pollinators via exposure to contaminated pollen and nectar. This observational study related variation in on-farm insecticide use behavior by different growers to pest abundance and insecticide residues in plant tissues. Our goal was to identify pest management practices that effectively control pests while minimizing hazards to pollinators under realistic agricultural conditions. Specifically, we addressed the following questions: (i) What is the “window of protection” against early-season pests provided by neonicotinoid insecticides applied at planting? (ii) How effective, and how necessary, are foliar insecticides applied in the mid-season for cucumber beetle control? And (iii) to what degree do insecticides used for pest management contaminate cucurbit flowers?

## Materials and Methods

### Study Systems

Our study was conducted in 2017 and 2018 on 62 cucurbit farms in the Upper Midwest, United States. These were comprised of 28 cucumber (*Cucumis sativus* L.) farms in Michigan, 18 pumpkin (*Cucurbita pepo* L.) farms in Ohio, and 16 watermelon (*Citrullus lanatus* [Thumb.] Matsum. & Nakai) farms in Indiana (see Bloom et al. 2021a for a map of the study sites). All study sites were commercial-scale working farms where the use of pesticides and their timing of application reflect typical management regimes. In cucumber and pumpkin, growers exclusively used seeds treated with FarMore FI400, which combines 3 fungicides (mefenoxam, fludioxonil, and azoxystrobin) and the neonicotinoid insecticide thiamethoxam using the Cruiser 5FS formulation (0.25–0.75 mg per seed). In pumpkin, growers direct-seeded into the field, except for a single farm in each year where growers transplanted seedlings they propagated from treated seeds. In watermelon, growers transplanted seedlings that were propagated from untreated seeds. During transplant, nearly half of the growers applied the neonicotinoids thiamethoxam (4 farms) or imidacloprid (5 farms) via soil drench. No foliar insecticides were applied in cucumber, whereas 12 pumpkin and 13 watermelon farms applied varying numbers of foliar sprays consisting of diverse insecticide chemistries in at least 1 yr, including 5 watermelon farms that applied the neonicotinoid acetamiprid via foliar sprays (see Table 1). The majority of farms were supplemented with managed honey bees and/or bumble bees for pollination, while also benefiting from a diversity of wild bee visitors (Bloom et al. 2021a).

**Table 1.** Details regarding chemical applications across 3 cropping systems

Crop (year)	No. farms	Treatment at planting/transplanting (no. farms)	Foliar insecticides (no. farms)
Cucumber (2017)	16	FarMore FI400 seed (16)	None applied
Cucumber (2018)	14	FarMore FI400 seed (14)	None applied
Pumpkin (2017)	14	FarMore FI400 seed (14) and imidacloprid drench (1)	Avermectins, carbamates, and pyrethroids (9)
Pumpkin (2018)	16	FarMore FI400 seed (16) and imidacloprid drench (1)	Carbamates and pyrethroids (9)
Watermelon (2017)	15	Thiamethoxam drench (4) and imidacloprid drench (4)	Avermectins, butenolides, neonicotinoids, organofluorines, organophosphates, pyrazoles, pyrethroids, and ryanoids (11)
Watermelon (2018)	15	Thiamethoxam drench (3) and imidacloprid drench (4)	Avermectins, butenolides, neonicotinoids, pyrazoles, pyrethroids, and ryanoids (11)

### Cucumber Beetle Pest Surveys

At all sites, we surveyed cucurbit plants for striped and spotted cucumber beetles (hereafter referred to collectively as “beetles”) over the crop growth cycle (see Table 2). Beetle surveys began when the seedlings’ first or second true leaf became fully expanded, and extended into the crop bloom period. During each survey at each site, we counted the number of beetles occurring on 20 focal cucurbit plants. In the late season, our ability to clearly delineate individual plants was often hindered as vines of adjacent plants intertwined with one another. When this occurred, we designated a 1-m^2^ patch around the center of each selected focal plant, yielding a somewhat conservative measure of the number of beetles per plant in cases where the total area of individual plants exceeded 1 m^2^. Focal plants were evenly spaced across 5 linear transects that ran perpendicular to the field margin and divided each field into roughly equal portions. Distance between neighboring focal plants along a transect was 5–75 m, depending on field size. Consecutive surveys at each site were generally separated by 1–3 wk.

**Table 2.** Details regarding surveys of cucumber beetles, cucurbit leaf tissue and flower tissue across 3 cropping systems

Crop (year)	No. beetle + leaf surveys	Beetle + leaf survey dates	No. flower surveys	Flower survey dates	No. flowers per sample
Cucumber (2017)	4–6	08 Jun–24 Aug	2	11 Jul–27 Aug	250–300
Cucumber (2018)	4–6	08 Jun–20 Aug	2	02 Jul–20 Aug	250–300
Pumpkin (2017)	4	19 Jun–04 Sep	2	19 Jul–04 Sep	60–80
Pumpkin (2018)	4–5	15 Jun–05 Sep	2	14 Jul–05 Sep	60–80
Watermelon (2017)	5–6	30 May–16 Aug	2	11 Jul–20 Aug	150
Watermelon (2018)	5–6	21 May–14 Aug	2	05 Jul–28 Aug	150

### Leaf and Flower Samples

During each beetle survey, we collected the youngest fully expanded leaf from the same 20 focal plants on which we counted beetles to evaluate the concentration of neonicotinoids. Leaves were placed in a sterile plastic bag in a cooler immediately upon collection and stored at −80°C within 24 h. We also collected floral tissue twice each year during peak bloom (see Table 2) to evaluate the concentration of insecticide residues present therein. Due to the difficulty of obtaining sufficient pure pollen grains for insecticide residue analysis, we collected pollen-laden synandria (i.e., fused anthers) instead (see Nixon 2014), while acknowledging that differential translocation of chemicals may cause their concentrations to vary between the pure pollen that bees collect and synandrium tissue itself. As floral size and morphology differed among the 3 crops, different strategies were employed to collect sufficient floral tissue to yield 1–3 g of pollen-laden synandria (Table 2). In all cases, we collected flowers directly into a cooler in the early morning before bees have removed significant quantities of pollen; synandria were extracted from flowers and stored at −80°C within 48 h.

In pumpkin, the much larger flowers allowed for collection of nectar for insecticide residue analysis. We collected flowers with nectar wells that had not yet opened for pollinator access, enabling us to open the nectar wells after collection to extract the secreted nectar. Nectar was collected from flowers within 6 h of collection using a 50-μl micropipette tip glued onto the needle adapter of a 5-ml syringe (a clean apparatus was used for each site), placed in 1.5-ml centrifuge tubes (pooling across all flowers within a site), and stored at −80°C within 12 h of collection.

### Measuring Insecticide Residues in Cucurbit Tissue

To quantify insecticide residues in cucurbit leaf and floral tissues, we extracted residue from samples using a modified QuEChERS protocol (Anastassiades et al. 2003, see also Bloom et al. 2021a). To prepare for extraction, we cut thin, latitudinal slices from the latitudinal median of each stack of 20 leaves gathered from each site during each survey to collect 1 g of leaf tissue, which we then homogenized using a bead beater homogenizer with 2 ml of ddH_2_O. Preparation of pollen-laden synandria varied across crops. For cucumber and watermelon, we analyzed 1 g and 3 g of pollen-laden whole synandria, respectively. For pumpkin, which had much larger synandria, we collected 2.5 g of anther tissue peeled off from the synandrium surface, as well as 0.5 g of pure pollen, for each 3-g analytical sample. Synandria from all 3 crops were homogenized within 50-ml centrifuge tubes using a sterile pestle. Pumpkin nectar was subjected directly to extraction as 1-ml samples after centrifuging to remove impurities. Extracted pesticide residue samples were analyzed using liquid chromatography and tandem mass spectrometry (LC-MS/MS) at Purdue University’s Bindley BioScience Center. Extraction and analysis protocols are reported in Supplementary Appendix S1.

For cucurbit leaves, we quantified concentrations of the 2 neonicotinoids applied by growers at planting: imidacloprid and thiamethoxam. For pumpkin synandria tissue and nectar, we additionally quantified concentrations of carbaryl, which was the only foliar insecticide that was both amenable to our chemical residue quantification protocol and applied at enough sites to warrant analysis. We calculated the concentrations of neonicotinoid insecticides by comparing against deuterated internal standards added to each analytical sample. We calculated carbaryl concentrations by comparing against a standard curve generated via 8 serial dilutions of a pesticide stock mixture made using analytical standards (see Bloom et al. 2021a). All quantifications were performed using Agilent MassHunter Quantitative Software B.09.00.

### Statistical Analyses

We performed all analyses in R version 3.6.3 (R Core Team 2023). Except where noted otherwise, all models included study year (2017 vs. 2018) as a categorical fixed-effect covariate to account for environmental differences between years, and farm identity as a random-intercept effect to account for repeated sampling. We analyzed insecticide concentration as log_10_(*x* + 0.1)-transformed parts per billion (ppb) to improve data normality. Sample sizes (the number of farms involved in each analysis) are reported in Table S1 of Supplementary Appendix S1.

#### Defining the “Window of Protection” Provided by Systemic Insecticides

To define the “window of protection,” that is, the duration of protection provided to cucurbit plants by systemic insecticides applied at planting, we quantified the rate at which insecticide concentration declined and the corresponding beetle abundance on cucurbit plants. To examine how concentrations of thiamethoxam and imidacloprid in leaf tissue declined through the growing season, we constructed linear mixed-effects models (LMMs) using R package *lme4* (Bates et al. 2015). One LMM was constructed for each neonicotinoid-crop combination to test how neonicotinoid concentration in leaf tissue varied with the number of days elapsed since planting. Only data from farms where growers applied the neonicotinoid of interest were included. We constructed thiamethoxam models for all 3 crops, and an imidacloprid model only for watermelon as no cucumber farm and only 2 pumpkin farms applied imidacloprid. To compare rates of decline across crops, we constructed an additional LMM that combined data from all models described above. Here, the independent variables were the number of days elapsed since planting, the identity of the neonicotinoid-crop combination, and their interaction. We used package *lmerTest* (Kuznetsova et al. 2017) to extract *P*-values for the fixed effects and package *MuMIn* (Bartoń 2016) to calculate marginal and conditional *R*^2^ values. For the additional LMM that included data from all crops, we also used package *emmeans* (Lenth 2020) to perform post hoc pairwise comparisons of the slopes of attenuation curves.

To test how beetle abundance varied in response to neonicotinoid concentrations in leaf tissue collected during the same survey, we constructed negative binomial, generalized additive models for location, scale, and shape (GAMLSSs) using package *gamlss* (Rigby and Stasinopoulos 2005). In watermelon, where >99% of cucumber beetles detected were *A. vittatum*, we modeled only that species; whereas in pumpkin, where both species co-occurred, we pooled them into a single count as a metric of the overall beetle pressure experienced (see also Yao et al. 1996). Despite the fact that the 2 beetle species have different life histories in our region, both attack cucurbit crops and respond readily to treatment by neonicotinoids (McLeod 2006). No model was constructed for cucumber, as only 4 beetles were detected across all farms.

One GAMLSS was constructed for each neonicotinoid-crop combination. Each model had a log-link function for *μ* (mean) and *σ* (variance) coefficients; models for thiamethoxam additionally included the coefficient *ν* (probability of zeroes), modeled with a logit-link function (Rigby and Stasinopoulos 2005), due to many surveys detecting no beetles. To isolate the impact of neonicotinoids, data points where growers had applied other insecticides were excluded from their respective models—for example, if a farm had a different insecticide applied after our second survey, then its data from the third survey onward were excluded.

#### Efficacy of Systemic and Foliar Insecticides for Beetle Control

To examine how insecticide management regimes affected the buildup of beetles as the growing season progressed, we constructed a negative binomial GAMLSS each for pumpkin and watermelon. Both models had *μ*, *σ*, and *ν* coefficients configured as described in the previous section. The pumpkin model tested how beetle abundance varied with foliar insecticide application status (applied vs. not applied) at the time of sampling, the number of days elapsed since planting, and their interaction. For watermelon, in which systemic insecticide application strategies varied across farms, the model tested how beetle abundance varied with foliar insecticide application status, the number of days elapsed since transplanting, and the application status of thiamethoxam. In the watermelon model, we did not include imidacloprid because initial models showed that its application status was unrelated to beetle abundance, and we did not include interaction terms among independent variables because doing so failed to improve model fit.

#### Hazard of Systemic and Foliar Insecticides to Cucurbit Pollinators

To ascertain whether insecticides contaminate floral resources, we constructed LMMs to test how insecticide concentration in synandria varied with application status. One model was constructed for each insecticide-crop combination that exhibited sufficient variation in application status: imidacloprid and carbaryl in pumpkin, and imidacloprid and thiamethoxam in watermelon (all cucumber growers used thiamethoxam-treated seeds and no foliar insecticides). Since the timing of carbaryl foliar application varied across pumpkin farms, we constructed an additional LMM to test how carbaryl concentration in synandria varied with the number of days elapsed since the most recent application. This latter model only included data points from farms where carbaryl had been applied earlier in the same year.

To compare potential hazards to pollinators from insecticide exposure across crops, we calculated a pollen hazard quotient (PHQ; Stoner and Eitzer 2013) for each insecticide in each crop by dividing its concentration in the synandria by its acute oral median lethal dose (LD_50_) for honey bees, obtained from the Pesticide Properties DataBase (University of Hertfordshire, http://sitem.herts.ac.uk/aeru/ppdb/en/index.htm). PHQs therefore provide a relative means through which to compare concentrations of different agrochemicals in light of their differing toxicity levels from the pollinator’s perspective. We then constructed an LMM to test how PHQ of insecticides in synandria varied across insecticide-crop combinations. Post hoc pairwise comparisons were performed using a Tukey adjustment for multiple comparisons.

Lastly, since pollinators consume different ratios of pollen and nectar, we constructed LMMs to examine whether insecticide concentrations in synandria are indicative of those in nectar. This analysis was limited to pumpkin, the only crop from which we could extract a sufficient volume of nectar. A separate model was constructed for each insecticide, wherein each data point consisted of insecticide concentration in a nectar sample (dependent variable) and synandria sample (independent variable) collected from the same flowers. To compare across insecticides with respect to the slope of the relationship described above, we constructed an additional LMM that included data for all insecticides, in which dependent variables included insecticide concentration in synandria, insecticide identity, and their interaction.

#### Tradeoffs Between Leaf Protection and Floral Resource Contamination by Systemic Insecticides

We constructed a linear model (LM) for each systemic insecticide-crop combination to test relationships between insecticide concentrations in synandria and temporally matched leaf tissue. We did not use LMMs in this case because models failed to converge when farm identity was included as a random effect. Thus, we instead constructed LMs with and without the inclusion of farm identity as an additional independent variable, and when both model structures yielded qualitatively similar results, we chose the simpler model (i.e., without the inclusion of farm identity), which had a lower AIC score. Each data point consisted of paired synandria and leaf tissue collected within 7 days of each other (usually the same day; mean temporal separation = 1.4 days); we excluded 13 watermelon and 6 pumpkin synandria samples that lacked corresponding leaf samples taken within 7 days.

For thiamethoxam, which was applied in all 3 crops, we constructed an additional LMM to determine whether the 3 crops varied with respect to the slope of the relationship described above. This additional model incorporates thiamethoxam data from all crops and is structured similarly to the models described above, except (i) farm identity was included as a random effect, and (ii) crop identity and its interaction with foliar thiamethoxam concentration were included as additional independent variables.

## Results

### Defining the “Window of Protection” Provided by Systemic Neonicotinoids

**Fig. 1.**
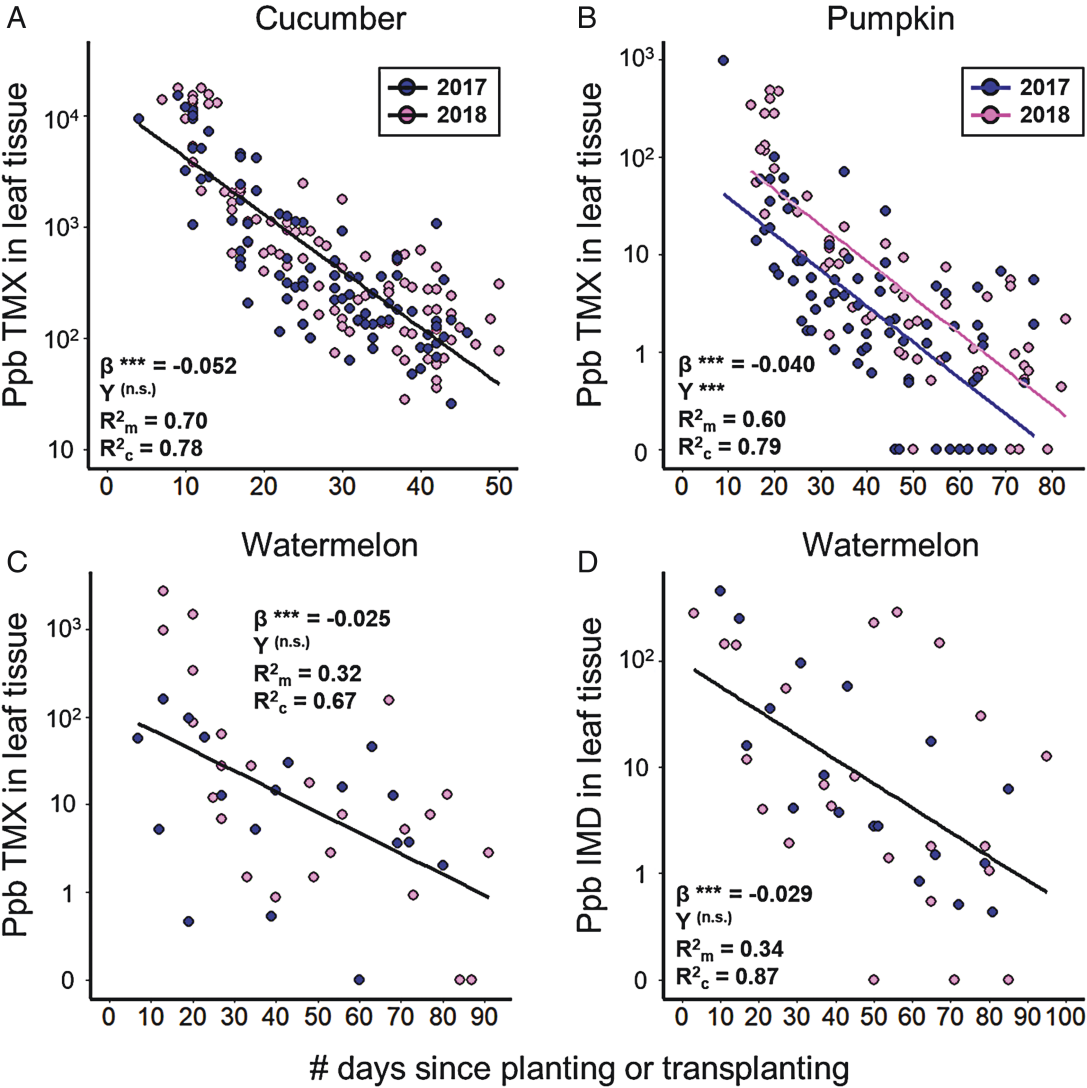
Assessing the “window of protection” provided by systemic neonicotinoid insecticides by quantifying declines in their concentration in cucurbit leaf tissue over time. Panels show (A) thiamethoxam (“TMX”) in cucumber, (B) thiamethoxam in pumpkin, (C) thiamethoxam in watermelon, and (D) imidacloprid (“IMD”) in watermelon. Thiamethoxam was applied as a seed coating treatment in cucumber and pumpkin, whereas thiamethoxam and imidacloprid were applied as a soil drench when transplanting seedlings into the field in watermelon. Data from farms where growers did not apply the focal neonicotinoid were excluded from this analysis. Data points depicting 0 ppb had concentrations that were below our threshold for quantification. Slope estimates (“β”) as well as marginal and conditional *R*^2^ values are provided for the effect of the number of days elapsed since planting on neonicotinoid concentration. Significance levels are provided for β and for the effect of study year (“Y”; 2017 in light points and 2018 in dark points); *** represents *P* < 0.005. Regression curves are plotted using models constructed without random effects to aid visualization; separate curves for each year are plotted only when the effect of study year is significant. Details of model outputs are reported in Table S6 of Supplementary Appendix S1; enlarged versions of individual panels are in Fig. S3 of Supplementary Appendix S1.

Concentrations of systemic neonicotinoids decreased log-linearly over time across all 3 crops (Fig. 1A–D), being statistically significant for both thiamethoxam (LMM *F*_1,158_ = 541, *P* < 0.0001 for cucumber, Fig. 1A; *F*_1,115_ = 344, *P* < 0.0001 for pumpkin, Fig. 1B; and *F*_1,36_ = 37.3, *P* < 0.0001 for watermelon, Fig. 1C) and imidacloprid (*F*_1,35_ = 87.4, *P* < 0.0001 for watermelon, Fig. 1D). The slope of this negative relationship differed across neonicotinoid-crop combinations (interaction between number of days elapsed and neonicotinoid-crop combination: *F*_3,348_ = 19.7, *P* < 0.0001), being most negative in thiamethoxam in cucumber (Fig. 1A), followed by thiamethoxam in pumpkin (Fig. 1B), whereas watermelon had the least negative slopes in which thiamethoxam and imidacloprid did not differ from each other (Fig. 1C; post hoc pairwise tests are reported in Table S2 of Supplementary Appendix S1). Study year was unrelated to neonicotinoid concentration in all cases (*P* > 0.05) except for thiamethoxam in pumpkin, for which overall concentrations in 2018 were higher than those in 2017 (*F*_1,122_ = 35.7, *P* < 0.0001, Fig. 1B).

**Fig. 2.**
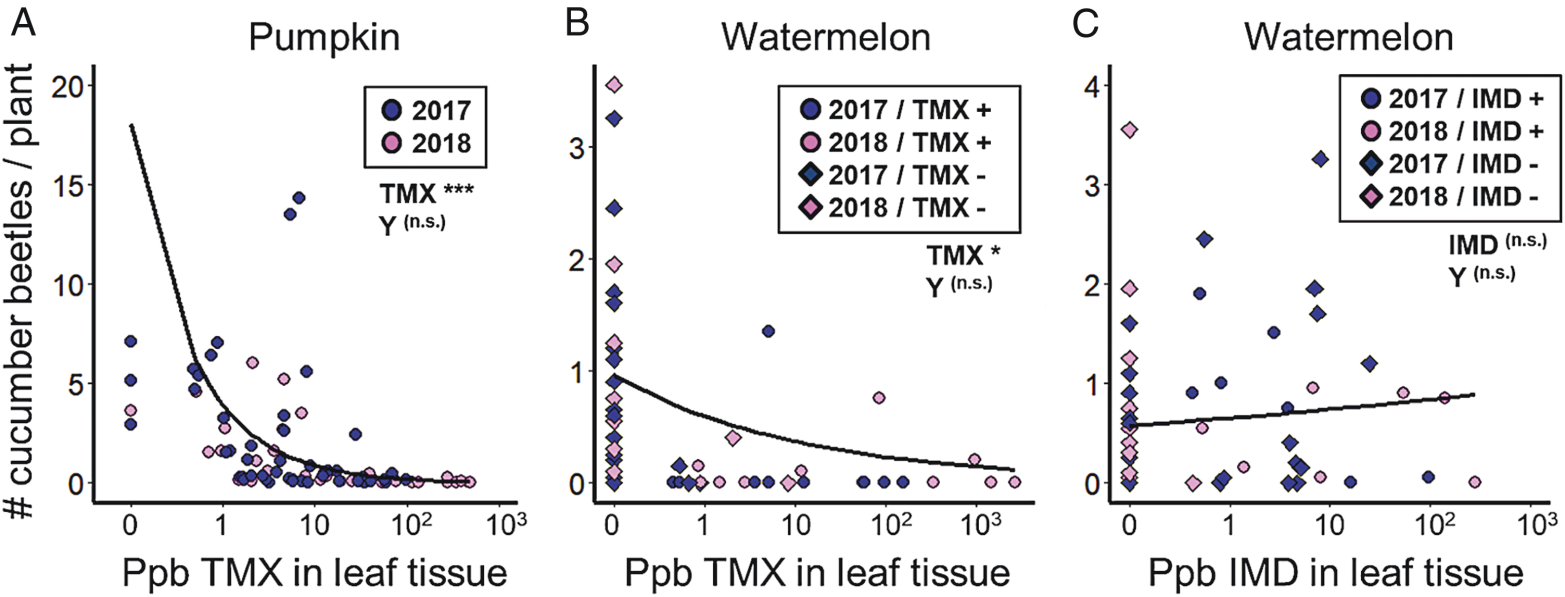
Relationships between cucumber beetle abundance and concentration of systemic neonicotinoid insecticides in cucurbit leaf tissue. Panels show (A) thiamethoxam (“TMX”) in pumpkin, (B) thiamethoxam in watermelon, and (C) imidacloprid (“IMD”) in watermelon. Data points where growers had applied insecticides other than the focal neonicotinoid were excluded from this analysis. Circles represent samples where growers had applied the focal neonicotinoid (i.e., as treated seeds or soil drench); diamonds represent samples where the focal neonicotinoid was not applied. Data points depicting 0 ppb had concentrations that were below our threshold for quantification. Significance levels are provided for the effect of neonicotinoid concentration on cucumber beetle abundance, and for the effect of study year (“Y,” 2017 in light points and 2018 in dark points); * represents *P* = 0.05, *** represents *P* < 0.005. Regression curves are plotted using models constructed without random effects to aid visualization. Details of model outputs are reported in Table S7 of Supplementary Appendix S1; enlarged versions of individual panels are in Fig. S4 of Supplementary Appendix S1.

Beetle counts were negatively associated with leaf tissue thiamethoxam concentrations in both pumpkin (negative binomial GAMLSS *t*_16,65_ = 12.4, *P* < 0.0001, Fig. 2A) and watermelon (*t*_6,60_ = 2.0, *P* = 0.051, Fig. 2B). In contrast, we found no evidence that imidacloprid controlled beetles in watermelon (*t*_11,47_ = 0.42, *P* = 0.68, Fig. 2C). Study year was unrelated to beetle abundance in all cases (*P* > 0.05).

### Efficacy of Systemic and Foliar Insecticides for Beetle Control

**Fig. 3.**
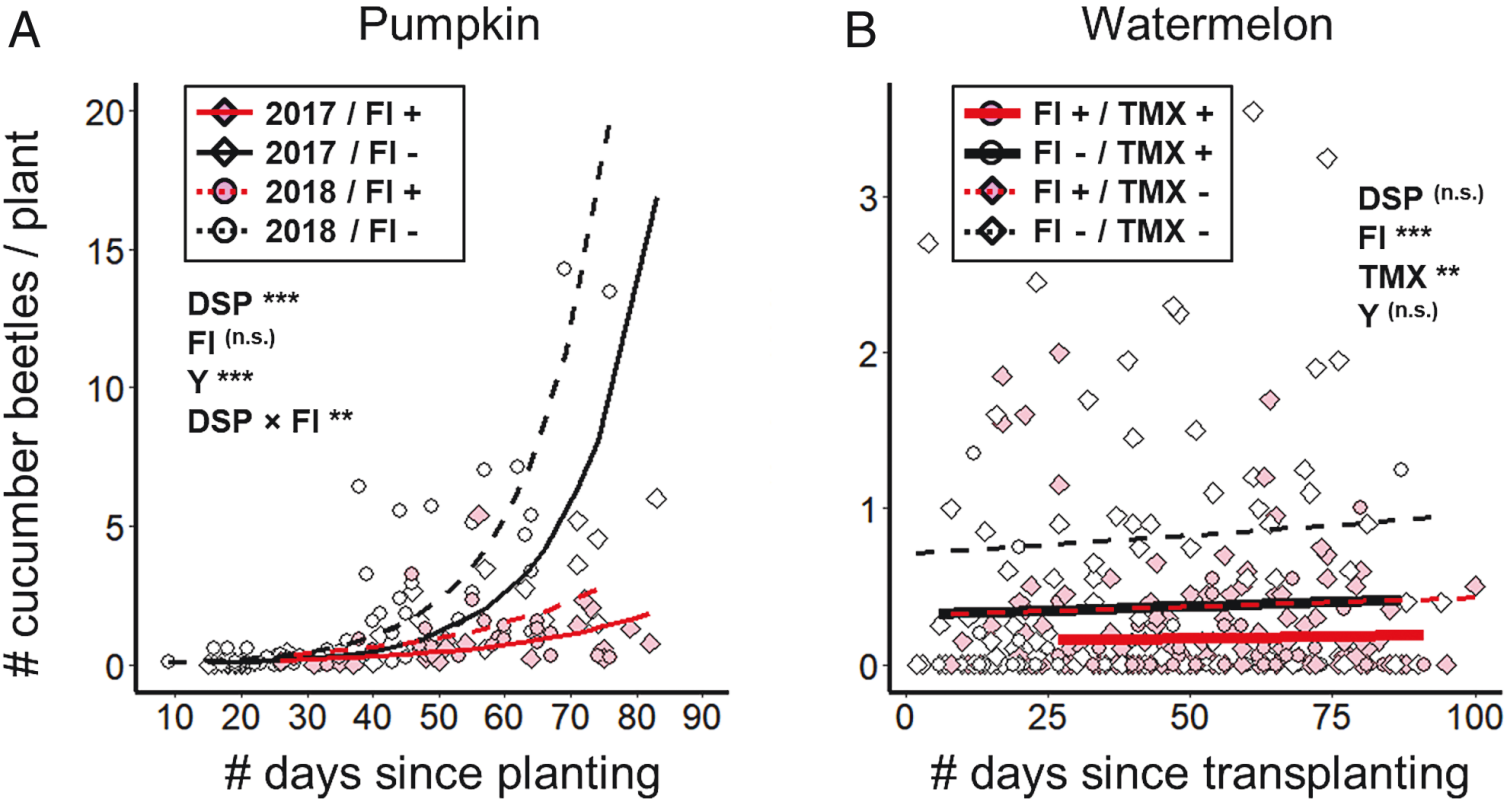
Effect of insecticide management regime on cucumber beetle buildup over time in (A) pumpkin fields and (B) watermelon fields through the growing season. In both panels, tinted points and regression lines represent samples where growers had applied a foliar insecticide (“FI”) prior to our sampling; unfilled points and black regression lines where no foliar insecticide had been applied prior to sampling. In panel (A), diamonds and solid lines represent data from 2017; circles and dashed lines represent data from 2018. In panel (B), circles and solid regression lines represent samples where growers applied thiamethoxam (“TMX”); diamonds and dotted regression lines where no thiamethoxam was applied (solid lines are thickened to aid visualization due to strong overlap between lines). Significance levels are provided for the effect of the number of days elapsed since planting on cucumber beetle abundance (“DSP”), and for the effects of foliar insecticide application status (“FI”), thiamethoxam application status, and study year (“Y”); ** represents *P* < 0.01, *** represents *P* < 0.005. Regression curves are plotted using models constructed without random effects to aid visualization; separate curves for each year are plotted only when the effect of study year is significant. Details of model outputs are reported in Table S8 of Supplementary Appendix S1; enlarged versions of individual panels are in Fig. S5 of Supplementary Appendix S1.

Application of insecticides contributed to beetle control throughout the growing season (Fig. 3A–B). In pumpkin, where beetle abundance increased over the growing season (negative binomial GAMLSS *t*_14,120_ = 10.6, *P* < 0.0001, Fig. 3A), the application of foliar insecticides significantly reduced the buildup of beetle abundance over time (interaction between foliar insecticide presence and number of days elapsed: *t*_14,120_ = 2.63, *P* = 0.01). Although the main effect of foliar insecticide application itself was not statistically significant (*t*_14,120_ = 1.63, *P* = 0.11), cucumber beetle abundances in farms not (yet) treated with foliar insecticides tended to increase sharply after the “window of protection” ended 4–6 wk after seeding, such that they regularly exceeded the economic threshold of 5 beetles per plant (Fig. 3A). In watermelon, which did not experience beetle buildup over time (*t*_7,274_ = 0.79, *P* = 0.43, Fig. 3B), and indeed never exceeded the economic threshold of 5 beetles per plant, beetle abundance was reduced by both foliar insecticides (*t*_7,274_ = 4.10, *P* < 0.0001) and systemic thiamethoxam (*t*_7,274_ = 3.21, *P* = 0.0015). Here, foliar insecticides and systemic thiamethoxam appeared to depress beetle abundances to similar extents and seemed to interact additively when both were applied (Fig. 3B). Beetle abundance was higher in 2018 than in 2017 in pumpkin (*t*_14,120_ = 4.38, *P* < 0.0001), but unrelated to study year in watermelon (*t*_7,274_ = 0.48, *P* = 0.63).

### Hazard of Systemic and Foliar Insecticides to Cucurbit Pollinators

**Fig. 4.**
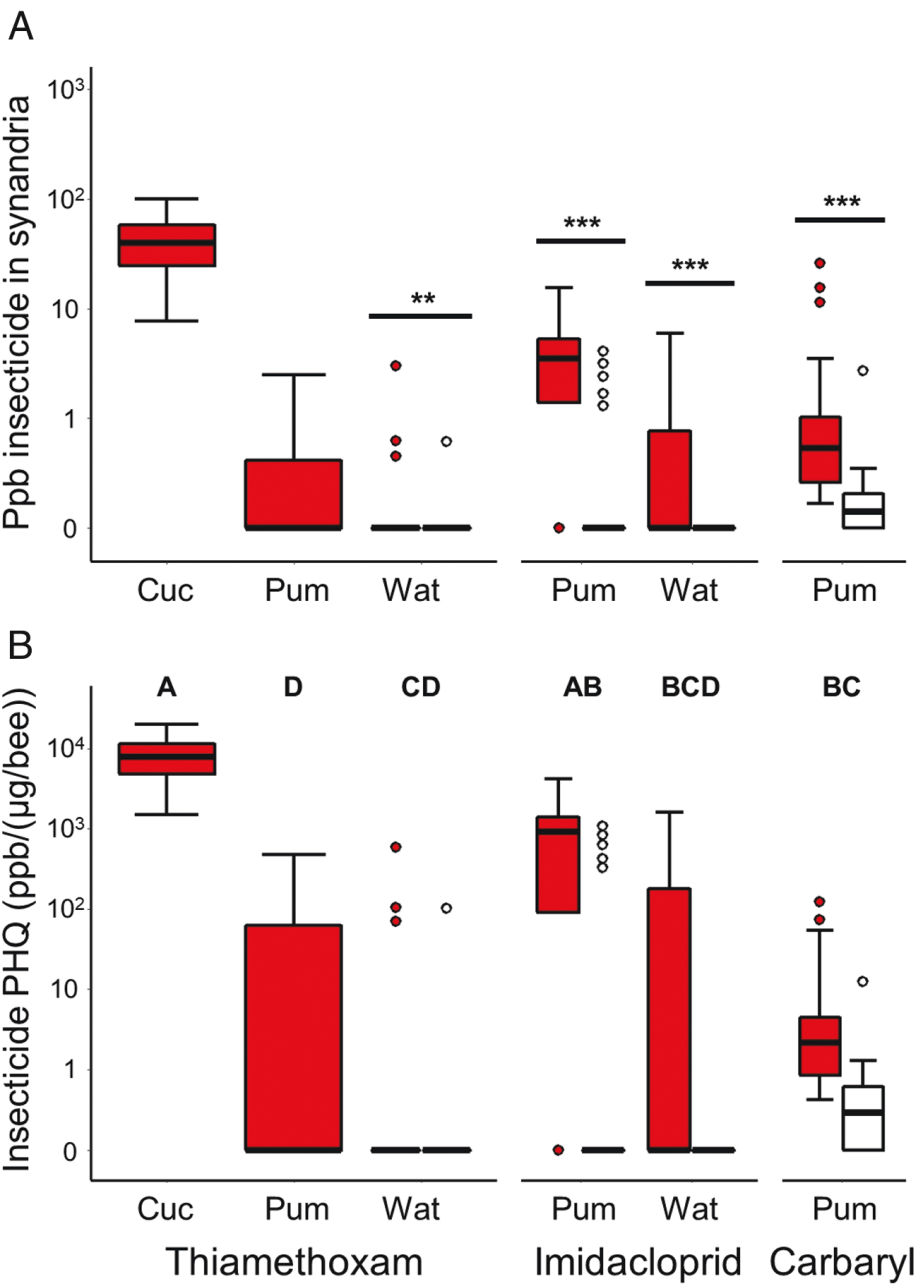
The (A) concentrations and (B) pollen hazard quotients (PHQ) of 3 insecticides in synandria of 3 cucurbit systems: cucumber (Cuc), pumpkin (Pum), and watermelon (Wat). Tinted boxes and points represent samples where the focal insecticide was applied; unfilled boxes and points represent samples where the focal insecticide was not applied. Boxes show central 50% of data, and bold horizontal lines represent the median; whiskers extend from the quartiles to 1.5× the interquartile range (or most extreme values of data, whichever is closest to median); points depict outliers, if any. Bars above pairs of boxes in (A) denote the statistical significance of comparisons between samples with versus without the focal insecticide applied; * represents *P* < 0.05, ** represents *P* < 0.01, and *** represents *P* < 0.005. Different letters above each set of boxes in panel (B) indicate insecticide-crop combinations that are statistically different from one another (*P* < 0.05) with respect to their PHQ; only data points where growers applied the insecticide in question were considered in this analysis.

Concentrations of systemic and foliar insecticides regularly exceeded detectable thresholds—as well as thresholds of concern (Hopwood et al. 2016)—in the synandria of cucurbit flowers (Fig. 4A). Applying an insecticide elevated its concentration in synandria for all tested insecticides: thiamethoxam (LMM *F*_1,18_ = 8.64, *P* = 0.009 in watermelon), imidacloprid (*F*_1,15_ = 19.4, *P* = 0.005 in pumpkin; *F*_1,52_ = 23.6, *P* < 0.0001 in watermelon), and carbaryl (*F*_1,19_ = 29.3, *P* < 0.0001 in pumpkin). We additionally found that concentrations in synandria decreased with time since application for carbaryl (*F*_1,10_ = 6.58, *P* = 0.028).

PHQs varied across crops and insecticides (Fig. 4B), with overall levels higher in 2018 than in 2017 (*F*_1,160_ = 6.99, *P* = 0.009). The highest PHQ was exhibited by thiamethoxam in cucumber synandria; thiamethoxam in pumpkin and watermelon exhibited the lowest PHQs. Imidacloprid and carbaryl, where applied, had intermediate values. Statistical details pertaining to pairwise comparisons across insecticide-crop combinations are reported in Table S3 of Supplementary Appendix S1.

**Fig. 5.**
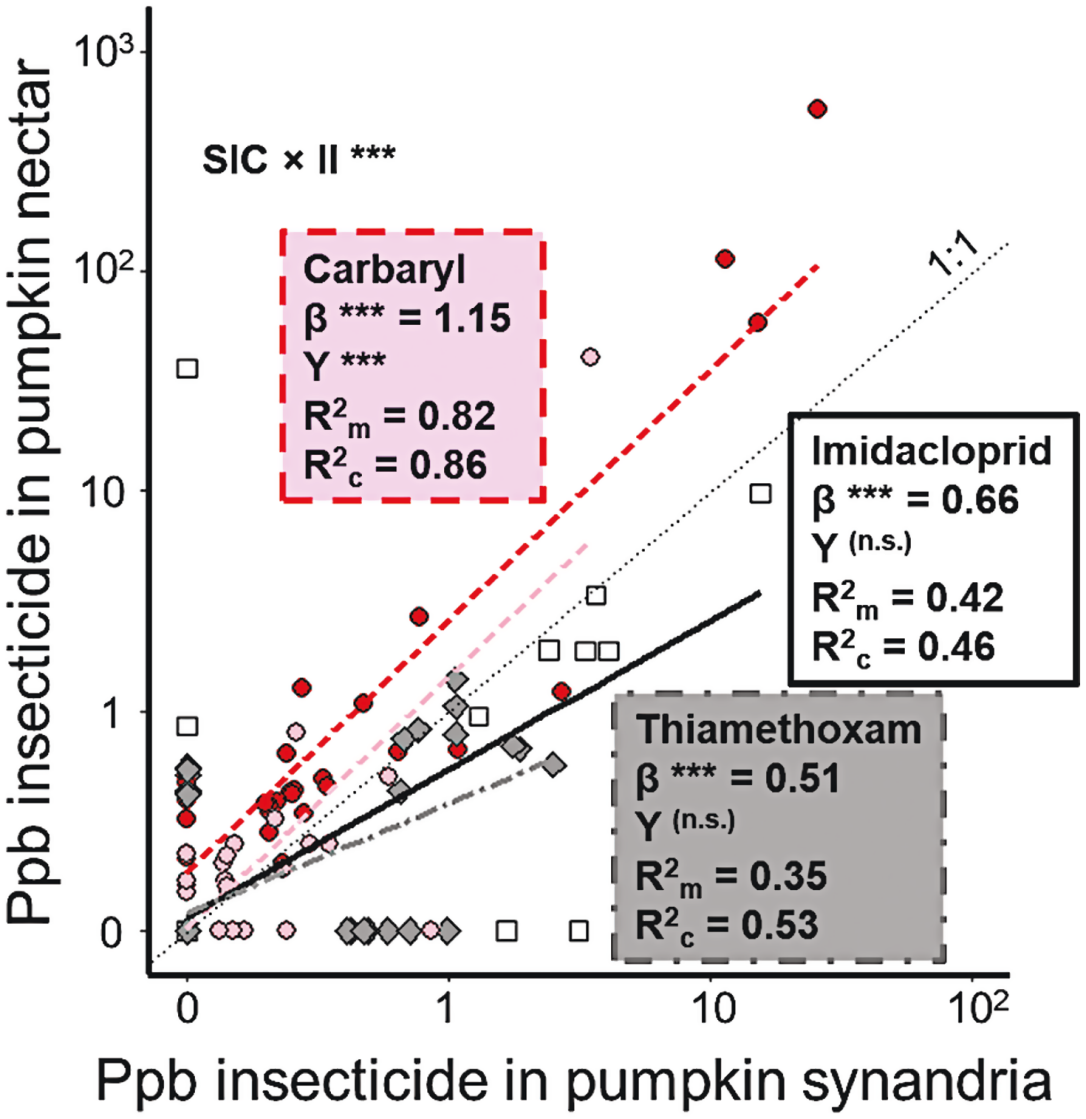
Concentrations of 3 insecticides in pumpkin synandria and nectar: carbaryl (dark circles and dashedline for 2017; light circles and dashed line for 2018), imidacloprid (squares and solid black line), and thiamethoxam (diamonds and dot-dashed line). The thin dotted line depicts a 1:1 relationship. Slope estimates (“β”) as well as marginal and conditional *R*^2^ values (*R*^2^_m_ and *R*^2^_c_, respectively) are provided for the effect of insecticide concentration in synandria on insecticide concentration in nectar. Data points depicting 0 ppb had concentrations that were below our threshold for quantification. Significance levels are provided for β and for the effect of study year (“Y”) for each insecticide’s individual model, as well as the interaction between synandria insecticide concentration and insecticide identity in the larger model comparing across insecticides (“SIC × II”); *** represents *P* < 0.005. Separate curves for each year are plotted only when the effect of study year is significant. Details of model outputs are reported in Table S9 of Supplementary Appendix S1. For visualization of each insecticide graphed separately, see Fig. S6 of Supplementary Appendix S1.

Insecticide concentrations in pumpkin nectar and synandria were positively related (Fig. 5) for carbaryl (LMM *F*_1,56_ = 186.9, *P* < 0.0001), imidacloprid (*F*_1,50_ = 41.3, *P* < 0.0001), and thiamethoxam (*F*_1,57_ = 33.4, *P* < 0.0001). Insecticide concentration in nectar was also higher in 2017 than in 2018 for carbaryl (*F*_1,52_ = 9.8, *P* = 0.003) but unrelated to study year for imidacloprid (*F*_1,50_ = 0.08, *P* = 0.79) and thiamethoxam (*F*_1,50_ = 1.31, *P* = 0.26). The 3 insecticides varied significantly with respect to the slope of the relationship described above (interaction between insecticide concentration in synandria and insecticide identity: *F*_2,173_ = 14.4, *P* < 0.0001; pairwise comparisons are reported in Table S4 of Supplementary Appendix S1).

### Tradeoffs Between Leaf Protection and Floral Resource Contamination by Systemic Insecticides

**Fig. 6.**
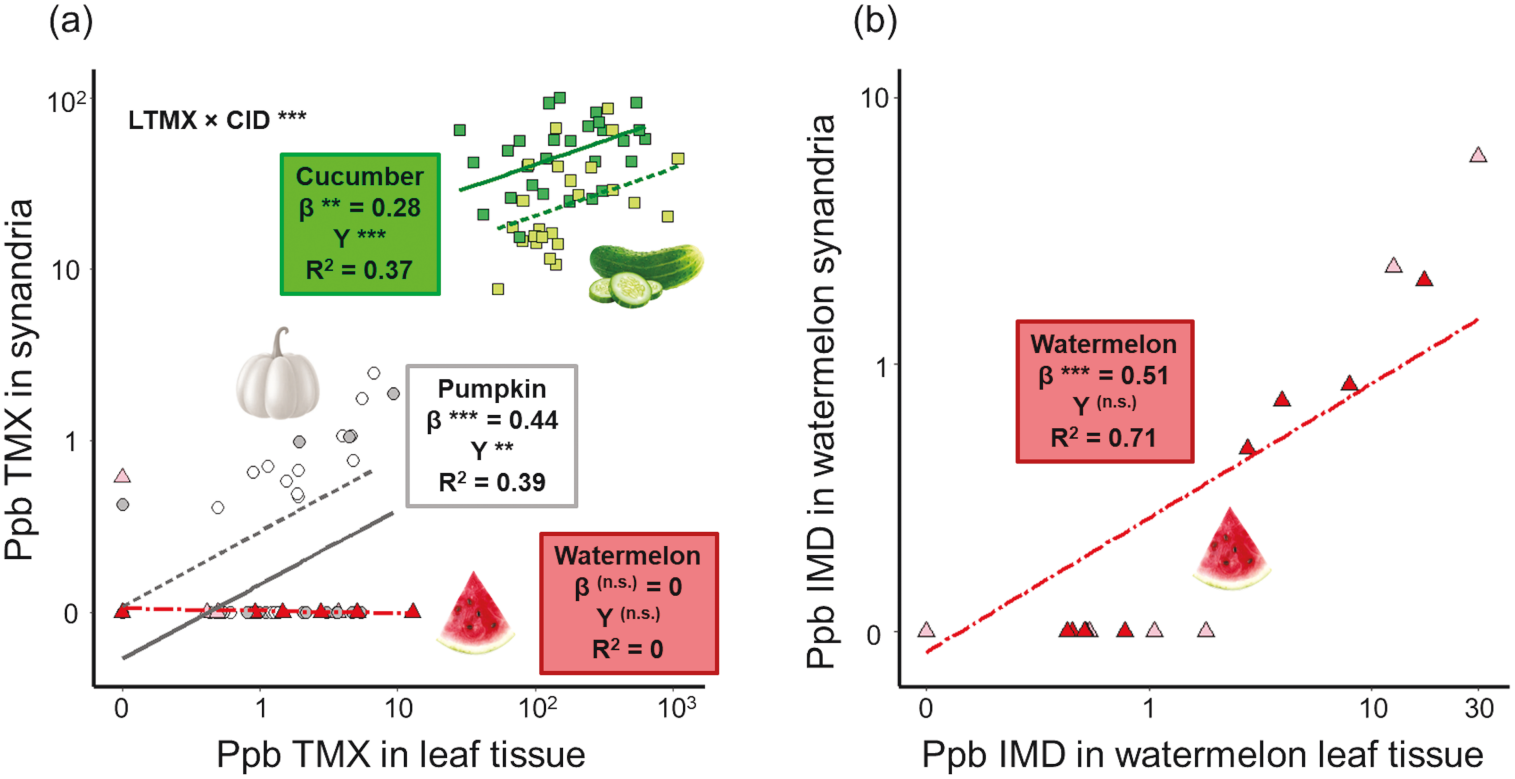
Concentrations of (A) thiamethoxam (“TMX”) and (B) imidacloprid (“IMD”) in temporally matched leaf and synandria of cucumber ( squares), pumpkin (circles), and watermelon (triangles). Data from 2017 are in darker shades and solid lines; data from 2018 are in lighter shades and dashed lines; dot-dashed regression lines represent both years of data. Data points depicting 0 ppb are those whose concentrations were below our threshold for quantification. Slope estimates (“β”) and *R*^2^ values are provided for the effect of neonicotinoid concentration in leaf tissue on that in synandria; significance levels are provided for β and for the effect of study year (“Y”) for each neonicotinoid’s individual model, as well as the interaction between leaf tissue thiamethoxam concentration and crop identity in the larger model comparing across crops (“LTMX × CID”) in panel (A); *** represents *P* < 0.005. Details of model outputs are reported in Table S10 of Supplementary Appendix S1. For visualization of each crop system in panel (A) graphed separately, see Fig. S7 of Supplementary Appendix S1.

Thiamethoxam concentrations in temporally matched leaf and synandria were significantly, positively correlated with each other (Fig. 6A) for cucumber (linear model *F*_1,23_ = 11.1, *P* = 0.003) and for pumpkin (*F*_1,33_ = 26.4, *P* < 0.0001), but not for watermelon (*F*_1,25_ = 0.16, *P* = 0.70). Synandria thiamethoxam levels were higher in 2017 than in 2018 for cucumber (*F*_1,23_ = 28.9, *P* < 0.0001) but higher in 2018 for pumpkin (*F*_1,33_ = 10.0, *P* = 0.003) and did not differ across study years for watermelon (*F*_1,25_ = 0.83, *P* = 0.37). The 3 crops varied with respect to the slope of the relationship described above (interaction between crop identity and thiamethoxam concentration in synandria: *F*_2,83_ = 7.78, *P* = 0.0006; pairwise comparisons are reported in Table S5 of Supplementary Appendix S1). The concentration of imidacloprid in watermelon synandria was also significantly correlated with that in leaf tissue (Fig. 6B; *F*_1,25_ = 187.1, *P* < 0.0001), though again, unrelated to study year (*F*_1,25_ = 0.62, *P* = 0.44).

## Discussion

### Contribution of Systemic and Foliar Insecticides to Cucumber Beetle Management

Across 3 cucurbit crops, we found that insecticides, primarily the neonicotinoid thiamethoxam, contributed to cucumber beetle management. This pest suppression effect was clearest in cucumber, a virtually pest-free system in which we observed only 4 beetles across thousands of plants surveyed in 30 site-years. The lack of pest pressure in this system is consistent with the ca. 10,000 ppb of thiamethoxam in seedling cucumbers, 2 orders of magnitude greater than that found in pumpkin and watermelon seedlings. Unlike in cucumber, insecticide use in the other 2 crops followed an intermediate strategy, and the low-moderate insecticide concentrations were consistent with higher pest abundance, depending in part on time since application. When thiamethoxam concentration in leaf tissue exceeded 10 ppb, it suppressed beetle counts to < 5 and < 1 per plant in pumpkin and watermelon, respectively. This concentration corresponds to a window of protection of 4–6 wk after seeding for pumpkin and 5–8 wk after transplanting for watermelon, corroborating accounts by cucurbit growers and extension specialists in our region. Notably, this window of protection coincides with pest pressure on vulnerable seedlings exerted by overwintering striped cucumber beetles, which generally emerge prior to cucurbit planting season in our region (Foster 2016, Haber et al. 2021). Windows of protection reported for neonicotinoid seed treatments from other crops are comparable in duration but are not always as well synchronized with pest phenology (e.g., Seagraves and Lundgren 2012, Knight et al. 2015, Zhang et al. 2016, Alford and Krupke 2017, Tang et al. 2017).

Despite the comparable windows of protection offered by thiamethoxam for watermelon and pumpkin, implications for beetle management differ. In pumpkin, beetle populations quickly increased when seed treatments wore off at ca. 50 days post-planting, whereas in watermelon, beetles remained at low densities throughout the season regardless of insecticide strategy. As a result, beetles routinely exceeded the action threshold of 5 beetles per plant in pumpkin mid-season, prompting foliar insecticide applications, but never approached a density that would justify management in watermelon fields (compare *y*-axes in Fig. 3A vs. 3B). Given these differences, effectively managing beetles in pumpkin can generally be achieved with as few as 2 insecticide applications: an early season seed treatment and a mid-season foliar application (see Fig. S1 in Supplementary Appendix S1). In contrast, watermelon can likely be cultivated without insecticide inputs in many sites and years. Recently, Leach and Kaplan (2022) tested the effects of densities as high as 9 striped cucumber beetles per plant—nearly twice their economic threshold—without observing any associated watermelon yield loss. In cucumber, the growers were apparently familiar with the strong pest suppression rendered by the very high levels of thiamethoxam, such that no cucumber grower applied any foliar insecticides between seeding and harvest (Table 1). Even still, our findings regarding windows of protection in pumpkin and watermelon suggest that thiamethoxam levels in cucumber may be well in excess of optimal levels, as foliar thiamethoxam concentrations in cucumber remained well above 10 ppb over our entire study period (Fig. 1A).

Given the effectiveness of thiamethoxam, it was surprising that we found no evidence that imidacloprid, another systemically applied neonicotinoid, suppressed beetle abundance in either watermelon (Fig. 2C) or pumpkin (see Fig. S2 in Supplementary Appendix S1), even though it translocated into plant tissue in sufficient quantities to contaminate pollen at levels of concern (Fig. 4). This finding contradicts earlier reports on the efficacy of imidacloprid in beetle suppression and damage prevention in cucurbit crops (Fleischer et al. 1998, Mac Intyre Allen et al. 2001, McLeod 2006, Jasinski et al. 2009). 5 watermelon and 2 pumpkin growers used imidacloprid in at least 1 yr of the study, suggesting a perceived benefit, at least among some growers. Our results suggest that the use of imidacloprid for beetle control in cucurbit crops needs to be critically reevaluated and directly compared with thiamethoxam, which appears to offer stronger pest-suppressive properties in our system.

In addition to variation in the type of neonicotinoid applied, growers varied in their application method, which likely affected plant uptake and expression. This variation largely reflects crop-type differences with cucumber and pumpkin farms employing seed treatments and watermelon farms using soil drenches. While our study was not specifically designed to test application methodology, soil drenches appeared more variable in their outcome than seed treatments (Fig. 1), whereas seed treatments are designed to deliver precise quantities of insecticide to the crop. However, interannual variation we detected (e.g., Figs. 1B and 6A) suggests that even for seed treatments, the uptake and subsequent expression of systemic insecticides may be subjected to environmental effects such as variation in rainfall. We also found dramatic differences in thiamethoxam expression between cucumber and pumpkin, even though both used the same Cruiser 5FS formulation seed treatment, which delivers comparable amounts of insecticide (0.25–0.75 mg per seed). This discrepancy could reflect physiological differences in how the 2 crops absorb and metabolize thiamethoxam from their roots or intrinsic growth differences leading to dilution effects (i.e., cucumber plants are smaller than pumpkin and thus may concentrate the same amount of insecticide in less vegetative tissue).

### Potential for Non-Target Effects on Pollinators

Given the strong positive association between neonicotinoid concentrations in cucurbit leaves and flowers and our finding that concentrations in flowers greatly exceeded those known to cause sublethal or lethal effects on pollinators (10–100 ppb for thiamethoxam in cucumber flowers [Goulson 2013, Hopwood et al. 2016]), it seems likely that pest control exerted by neonicotinoids adversely affected wild pollinators. This pattern was most apparent in cucumber, where a companion study using the same farm sites reported that 98.4% of floral visitors were honey bees compared to > 50% of visitors consisting of wild bees observed in pumpkin and watermelon (Bloom et al. 2021a). These findings suggest that thiamethoxam seed treatments may have increased grower dependence on managed honey bees for pollination. At least 60 species of wild bees reportedly visit cucumber in the Upper Midwest region (Kauffeld and Williams 1972, Lowenstein et al. 2012, Smith et al. 2013), with honey bees representing 66%–81% of visits, where quantitative data are available, though determining a precise causal relationship requires focused study.

The high honey bee activity on cucumber blossoms in spite of high thiamethoxam levels could be related to the fact that honey bees on these farms primarily collect nectar (Wood et al. 2018), which accumulates lower neonicotinoid levels than does pollen (Goulson 2013), a pattern we also observed in pumpkin pollen and nectar (Fig. 5). Traits such as large body size and eusociality, combined with apiary management, may buffer honey bees from immediate negative effects of insecticide exposure (Arena and Sgolastra 2014, Heard et al. 2017) in high-thiamethoxam environments like cucumber fields. However, although managed honey bees may be shielded from cross-generational effects of pesticide exposure experienced by solitary wild bees (e.g., Stuligross and Williams 2021), prolonged exposure at the individual and colony level may still result in deleterious effects (Traynor et al. 2021, Tosi et al. 2022).

While insecticide applications in our fields led to higher leaf and flower concentrations (Fig. 4) as expected, we also detected residues in untreated plants. This outcome has been observed in related studies (Nixon 2014, Obregon et al. 2022) and should be anticipated given that cucurbits are typically rotated with corn and soybean, which similarly receive neonicotinoid seed treatments that can persist in the soil across years (Jones et al. 2014). Recent analyses indicate that such carryover effects could have consequences for wild pollinator conservation, especially ground-nesting bees (Willis Chan et al. 2019, Main et al. 2020).

Scouting fields for sporadic pest outbreaks can be an effective alternative to prophylactic neonicotinoids. In watermelon, this approach enhanced pollination, leading to higher yields (Pecenka et al. 2021, Leach et al. 2022). Unfortunately, IPM-based scouting approaches may sometimes cause greater harm than prophylactic neonicotinoids to beneficial insects when applying broad-spectrum foliar sprays (Rowen et al. 2022). In our system, the most common foliar insecticides used were carbaryl in pumpkin and pyrethroids (permethrin, bifenthrin, lambda-cyhalothrin) in watermelon, all of which are non-selective and highly toxic to bees, with at least carbaryl translocating into pollen and nectar (Figs. 4 and 5). For optimal balance of pest management and pollinator health, these products could be replaced with alternatives like acetamiprid, which provides excellent control of cucumber beetles with far lower hazard to bees (Nixon 2014).

### Caveats and Limitations

A major strength of our study is the use of large-scale commercial crop fields where growers determined inputs and application rates, such that the data represent “real” values experienced on working farms. A weakness of this approach is that the patterns are largely correlative, such that certain mechanisms can only be inferred from the data. For example, since all pumpkin and cucumber growers used seed treatments, we can only directly interpret the impacts of neonicotinoid use in watermelon where growers varied in use patterns. In pumpkin, the inverse relationship between declining neonicotinoid concentrations and rising beetle numbers over the growing season could be used as indirect evidence of thiamethoxam efficacy. However, this temporal correlation assumes that background beetle pressure is relatively constant over time. Given that striped cucumber beetle in the Midwestern United States has a large overwintering generation that coincides with cucurbit seedling emergence in late spring (Foster 2016), this assumption seems aligned with pest biology.

An additional limitation is that crop type is confounded by geography since each crop was studied in a different state. This choice was intentional since we focused on major economic hubs of production (e.g., Michigan is the top state in the United States for pickling cucumbers). However, this choice also makes it impossible to separate crop from state. For instance, it could be argued that the more northerly latitude of cucumber fields in Michigan, rather than the very high thiamethoxam concentrations, may have excluded beetles or delayed buildup. This alternative explanation seems unlikely though, as previous work in Michigan revealed appreciable beetle pressure (Haber et al. 2021), even in small-scale experimental plantings of susceptible (i.e., without thiamethoxam seed treatment) cucumber during the same seasonal timeframe as our study (Brandt 2012). Untreated cucumber plants, in general, are highly vulnerable to striped cucumber beetle infestations (Brandt 2012, Ingwell and Kaplan 2019, Kahl et al. 2019), and this pest also responds favorably to large cucumber monocultures in our study area (Bach 1980), so high foliar concentrations of thiamethoxam are very likely maintaining beetles at densities approaching zero.

### Implications for Integrated Pest and Pollinator Management

Prophylactic neonicotinoids (at least, thiamethoxam) can provide excellent control of cucurbit pests; however, given the strong association documented here and in related studies (Dively and Kamel 2012, Stoner and Eitzer 2012, Nixon 2014, Obregon et al. 2022) between vegetative and floral insecticide concentrations, evidence-based guidance is needed to avoid doing more harm than good, in terms of both ecological sustainability and economic profitability. Indeed, chronic exposure to even the lower range of PHQs detected in our study (Fig. 4B) may lead to significant sublethal effects for insect pollinators (reviewed in Tosi et al. 2022). Another way of framing this delicate balance is to ask, “how pest-free do crop fields need to be?” We show that cucumber exhibited extraordinarily high vegetative and floral thiamethoxam levels that resulted in virtually no pests—but that are simultaneously highly hazardous to pollinators. Even in watermelon, a system with far lower neonicotinoid levels than cucumber, inputs were poorly justified given pest levels relative to economic thresholds. Reducing management intensity by removing seed treatments and relying on traditional IPM-based scouting approaches, or reducing application rates coated onto seeds, would likely enhance system sustainability and profitability. In recent work for watermelon, we demonstrated that avoiding prophylactic applications and instead using scouting and pest thresholds to guide management resulted in cost savings from reduced chemical expenditures (Ternest et al. 2020) and higher yields from enhanced wild bee pollination (Pecenka et al. 2021), including on large commercial fields (Leach et al. 2022), some of which were included in this study.

Beyond these benefits, more data-driven insecticide use may reduce rental costs of honey bee hives. Some cucurbits could be pollinated exclusively by wild bees (as shown for pumpkin in Pennsylvania [McGrady et al. 2020]), but doing so may necessitate a strong reduction in chemical inputs. Although most wild pollinator research emphasizes the provisioning of natural habitat and alternative forage around crop fields, these efforts may fail without first addressing the toxicity of the environment (e.g., Wood et al. 2019, Ward et al. 2022).

It remains to be seen whether the varied economic benefits from implementing IPM counteract the potential complications and costs arising from pest scouting. Given the status quo, growers may be either satisfied with a risk-averse strategy, or unaware (or unconvinced) that the economic costs associated with insecticide use on their farms could outweigh the benefits of guarding against damage by pests (see sociological analysis of cucurbit growers in this region [Bloom et al. 2021b] and in other crops [Nalepa et al. 2020, Hevia et al. 2021]). If the latter, future studies that explicitly quantify the economic outcomes of different pesticide application strategies (e.g., Ternest et al. 2020) will be invaluable in helping entomologists and growers to collaboratively design implementable action plans that duly account for the contributions of wild pollinators to crop pollination and current threats to pollinator health, and make full use of scouting and other IPM-based tools on farms.

## Supplementary Data

Supplementary data are available at *Journal of Economic Entomology* online.

toae202_suppl_Supplementary_Materials

## Data Availability

Raw data included in this study are available via Figshare: https://figshare.com/articles/dataset/Study_raw_data/23739708
