# Supplementary material for: Plant versus pollinator protection: balancing pest management against floral contamination for insecticide use in Midwestern US cucurbits
Source: J Econ Entomol. 2024 Sep 15;118(1):262–73. doi: 10.1093/jee/toae202 (PMC11818373; doi:10.1093/jee/toae202)
Supplement: toae202_suppl_Supplementary_Materials [file toae202_suppl_supplementary_materials.pdf]

JOURNAL OF ECONOMIC ENTOMOLOGY

Plant vs. pollinator protection: Balancing pest management against floral contamination for insecticide use in Midwestern U.S. cucurbits

Keng-Lou James Hung*, John J. Ternest, Thomas J. Wood, Laura L. Ingwell, Elias H. Bloom, Zsofia Szendrei, Ian Kaplan, Karen Goodell

*Author for correspondence: kenglou.hung@gmail.com

APPENDIX S1. SUPPLEMENTAL METHODS, FIGURES, AND TABLES

Pesticide residue extraction and quantification

Neonicotinoid residues were extracted from leaf, pollen, and nectar samples using a modified QuEChERS (Quick-Easy-Cheap-Effective-Rugged-Safe) protocol (Anastassiades et al. 2003). For each leaf sample across all cropping systems, the 1-g homogenized leaf tissue was placed into a 15-ml centrifuge tube with 6 ml of extraction solution (2 ml of ddH2O plus 4 ml of acetonitrile (ACN)) and 10 µl of an internal standards solution containing d3-clothianidin (10 ng μl^{-1}), d4-imidacloprid (10 ng μl^{-1}) and d3-thiamethoxam (10 ng μl^{-1}). Samples from watermelon also included an internal standard for d3-acetamiprid (10 ng μl^{-1}). Samples were vortexed, and then 1.2 g of magnesium sulfate ($MgSO_4$) and 0.3 g of sodium acetate (NaOAc) were added. After vortexing and shaking, the tubes were centrifuged at 2500 rpm for 10 min, after which 1 ml of supernatant was transferred to a 2-ml dispersive Solid Phase Extraction tube containing 25 mg of primary secondary amine, 7.5 mg of Graphitized carbon black, and 150 mg of $MgSO_4$ (Agilent Technologies, Santa Clara, California, part number 5982–5321). Tubes were vortexed and then centrifuged at 15,000 RPM for 5 min. Next, the entire supernatant was transferred to a 1.5-ml centrifuge tube and dried completely in a speed vacuum. Prior to instrumental analysis, this sample was resuspended in 100 µl of ACN, centrifuged at 4000 rpm for 5 min, and then the entire volume was transferred to 96-well plates for analysis.

For each pollen sample across all cropping systems, the sample (1 g for cucumber, 3 g for pumpkin and watermelon) was placed into a 50-ml centrifuge tube with 30 ml of extraction solution (15 ml of ddH2O plus 15 ml of ACN) and 10 µl of the same internal standards solution used for leaf samples. Samples were vortexed, and then 6 g of $MgSO_4$ and 1.5 g of NaOAc were added. Tubes were vortexed and centrifuged at 2500 rpm for 10 min, after which 15 ml of supernatant was transferred to a 15-ml dispersive Solid Phase Extraction tube containing 400 mg of primary secondary amine, 400 mg of C18EC and 1200 mg of $MgSO_4$ (Agilent Technologies, part number 5982–5158). Tubes were vortexed and then centrifuged at 4000 rpm for 5 min. Next, 6 ml of supernatant was transferred to a 15-ml centrifuge tube and dried completely in a speed vacuum. Prior to instrumental analysis, this sample was resuspended in 1 ml of ACN, centrifuged at 4000 rpm for 5 min, and then 200 ml of the solution was transferred to 96-well plates for analysis.

For each pumpkin nectar sample, the 1-ml sample was placed into a 5-ml centrifuge tube with 3 ml of extraction solution (1 ml of ddH2O plus 2 ml of ACN) and 10 µl of the same internal standards solution used for leaf and pollen samples. Samples were vortexed, and then 300 mg of $MgSO_4$ and 75 mg of NaOAc were added. Tubes were vortexed and centrifuged at 2500 rpm for 10 min, after which 1 ml of supernatant was transferred to a 2-ml dispersive Solid Phase Extraction tube containing 50 mg of primary secondary amine and 150 mg of $MgSO_4$ (Agilent Technologies, part number 5982–5022). Tubes were vortexed and then centrifuged at 4000 rpm for 5 min. Next, the entire supernatant was transferred to a 1.5-ml centrifuge tube and dried completely in a speed vacuum. Prior to instrumental analysis, this sample was resuspended in 100 µl of ACN, centrifuged at 4000 rpm for 5 min, and then the entire volume was transferred to 96-well plates for analysis.

Pesticide residue analysis was performed using liquid chromatography (LC) and tandem mass spectrometry (MS) at the Bindley Bioscience Center at Purdue University, West Lafayette, Indiana. An Agilent Zorbax StableBond-Phenyl Column (2.1 x 100 mm, 3.5 µm; Agilent Technologies, PN 861753-912) was used for LC separation and an Agilent 1260 Infinity II LC system coupled to an Agilent 6470 series triple quadrupole mass spectrometer was used to identify pesticide residues based on retention time and co-chromatography with high-purity analytical standards. Analytical-grade standards were purchased from Sigma-Aldrich. This method allowed for the precise detection and identification of neonicotinoids present in samples down to concentrations of parts per billion, and the limits of quantification (LOQ) were 0.1–0.3 µg/L.

For carbaryl, a stock mixture that contained analytical standards for multiple insecticides and fungicides (including carbaryl) was prepared, and from this mixture, a series of 8 serial dilutions was created and analyzed on the instrument to establish a standard curve to which carbaryl residues in samples was calibrated to determine final concentrations (see also Long & Krupke, 2016). The LOQ for carbaryl was 720 µg/L. All processing of pesticide residue analysis data was performed in Agilent MassHunter Quantitative Software B.09.00.

References

Anastassiades M, Lehotay SJ, Štajnbaher D, Schenck FJ. 2003. Fast and easy multiresidue method employing acetonitrile extraction/partitioning and "dispersive solid-phase extraction" for the determination of pesticide residues in produce. Journal of AOAC International. 86(2):412–431. doi:10.1093/jaoac/86.2.412.

Long EY, Krupke CH. 2016. Non-cultivated plants present a season-long route of pesticide exposure for honey bees. Nature Communications 7:11629. doi:10.1038/ncomms11629.

Supplemental figures and tables

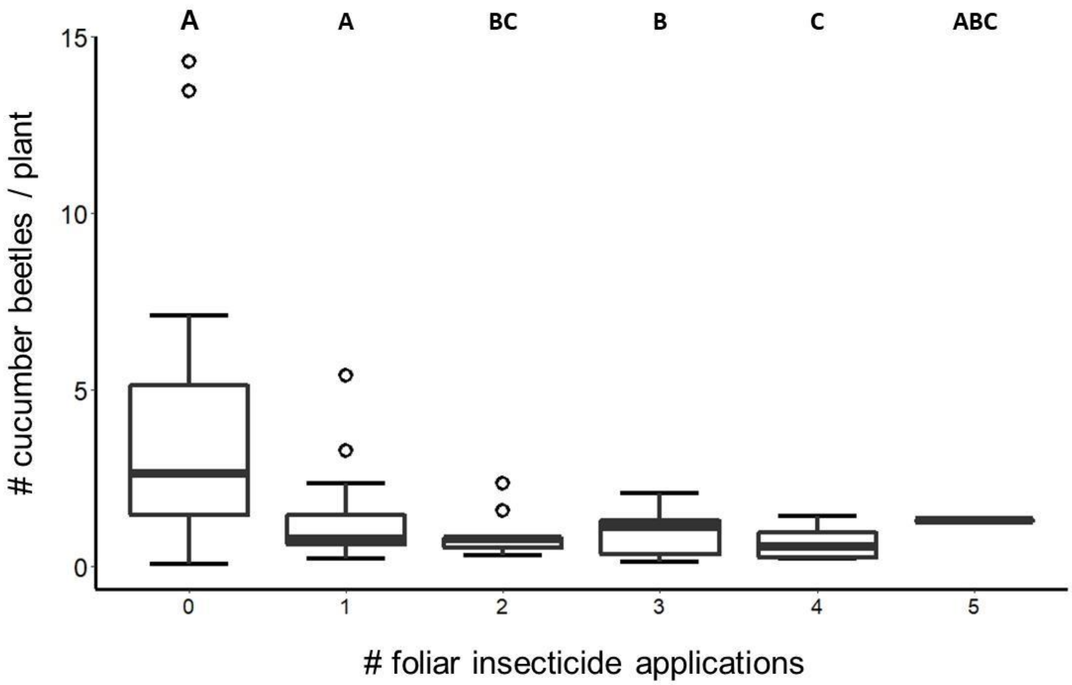

Figure S1: Control of cucumber beetles by foliar insecticides in pumpkin during the latter half of the growing season (i.e., > 40 days since planting) when systemically applied thiamethoxam offers limited protection. Boxplots and letters above boxes are as in Figure 4 of the main text. The cumulative number of foliar sprays performed at a site during the same growing season significantly influenced cucumber beetle abundances as revealed by a Poisson GLMM with cucumber beetle abundance as the dependent variable, the number of foliar sprays accumulated as a categorical factor, study year and the number of days elapsed since planting (scaled to a mean of 0 and standard deviation of 1 to aid model fitting) as covariates, and study site as a random effect.

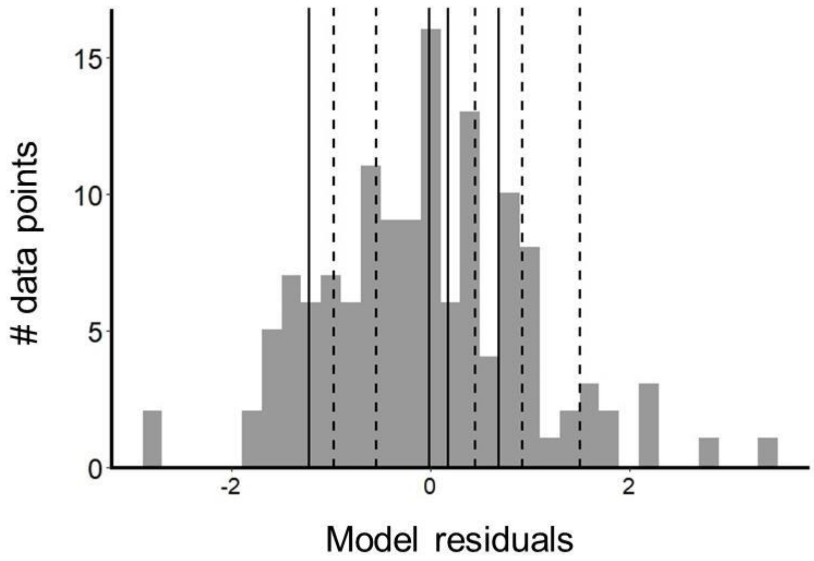

Figure S2: Histogram of model residuals of the analysis presented in Fig. 3a. Values of residuals of data from the two pumpkin farms that utilized systemically applied imidacloprid, one in each study year, are indicated in solid and dashed lines, respectively. Pumpkin farms that utilized systemically applied imidacloprid did not consistently experience enhanced cucumber beetle control (i.e., negative residuals) throughout the growing season relative to farms that did not utilize imidacloprid.

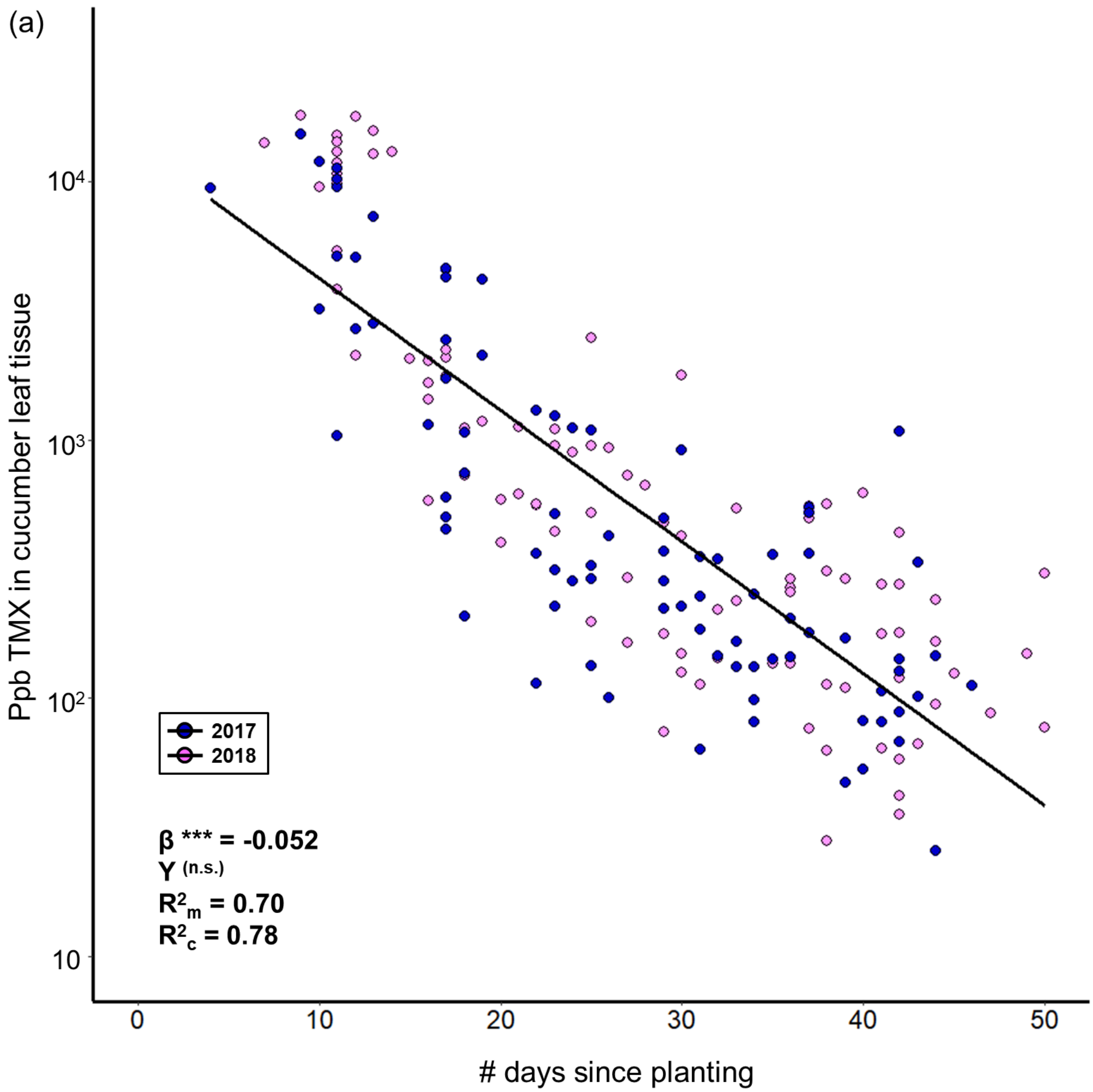

Figure S3a. Enlarged version of Figure 1a in the main text.

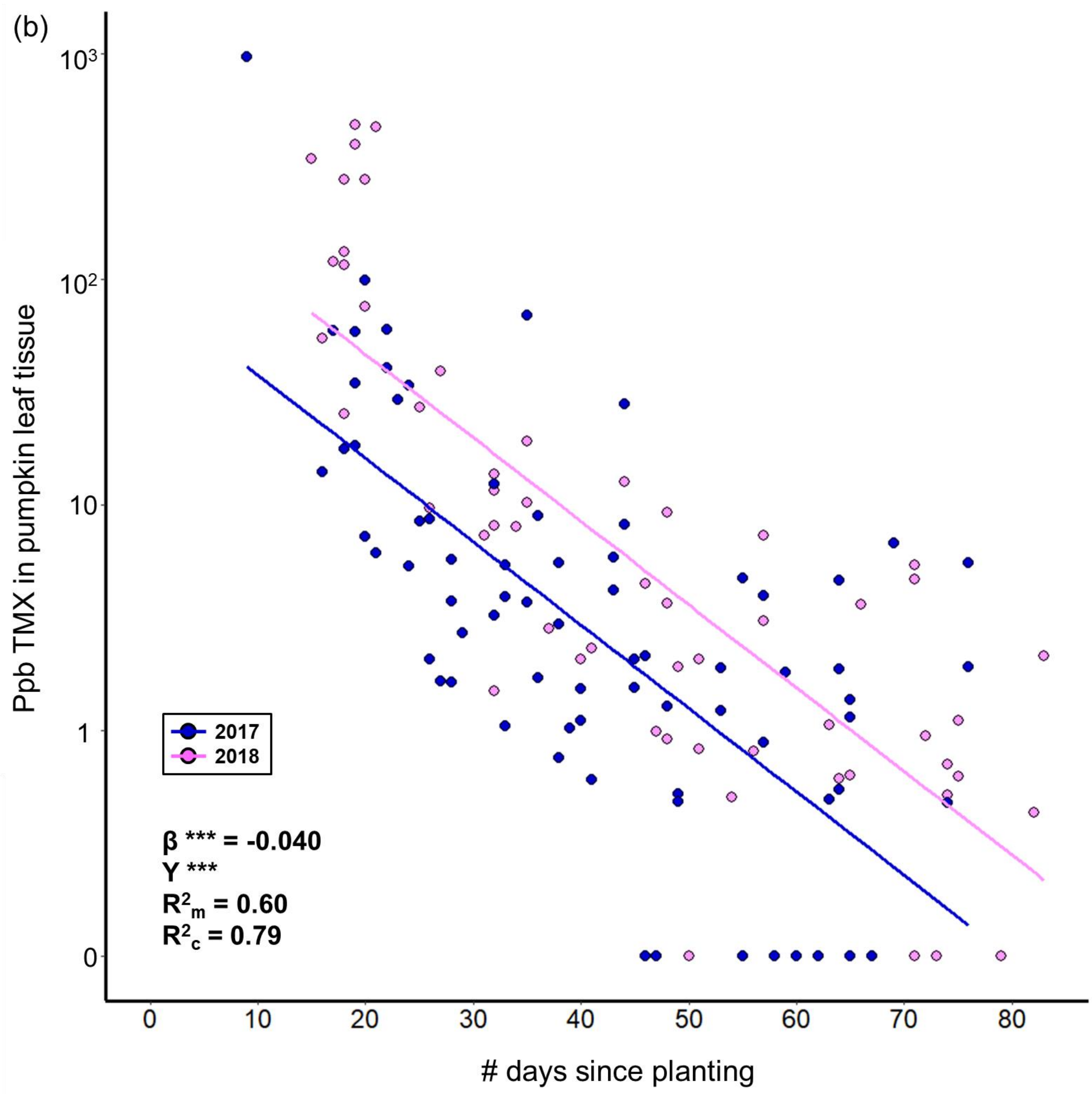

Figure S3b. Enlarged version of Figure 1b in the main text.

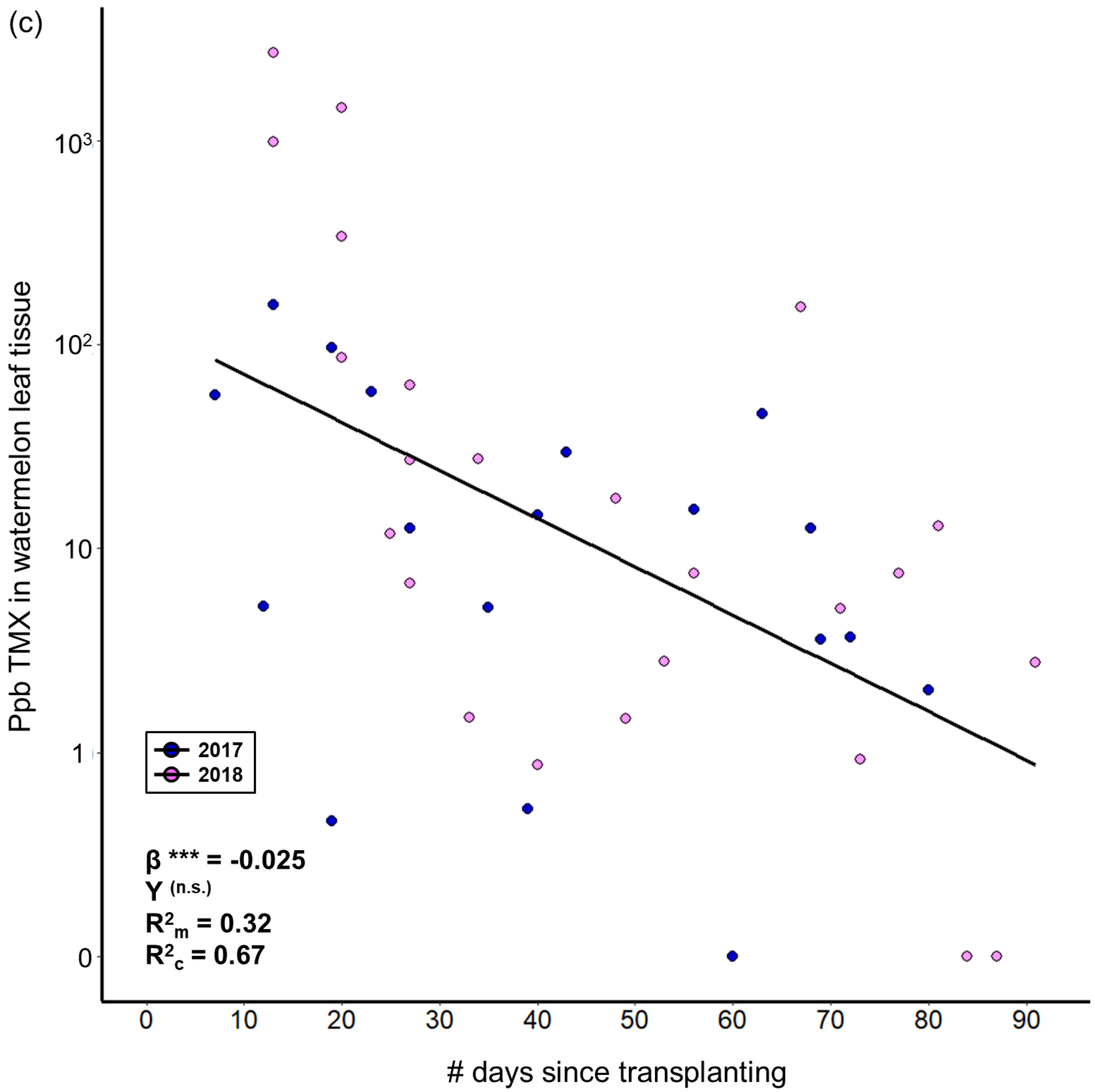

Figure S3c. Enlarged version of Figure 1c in the main text.

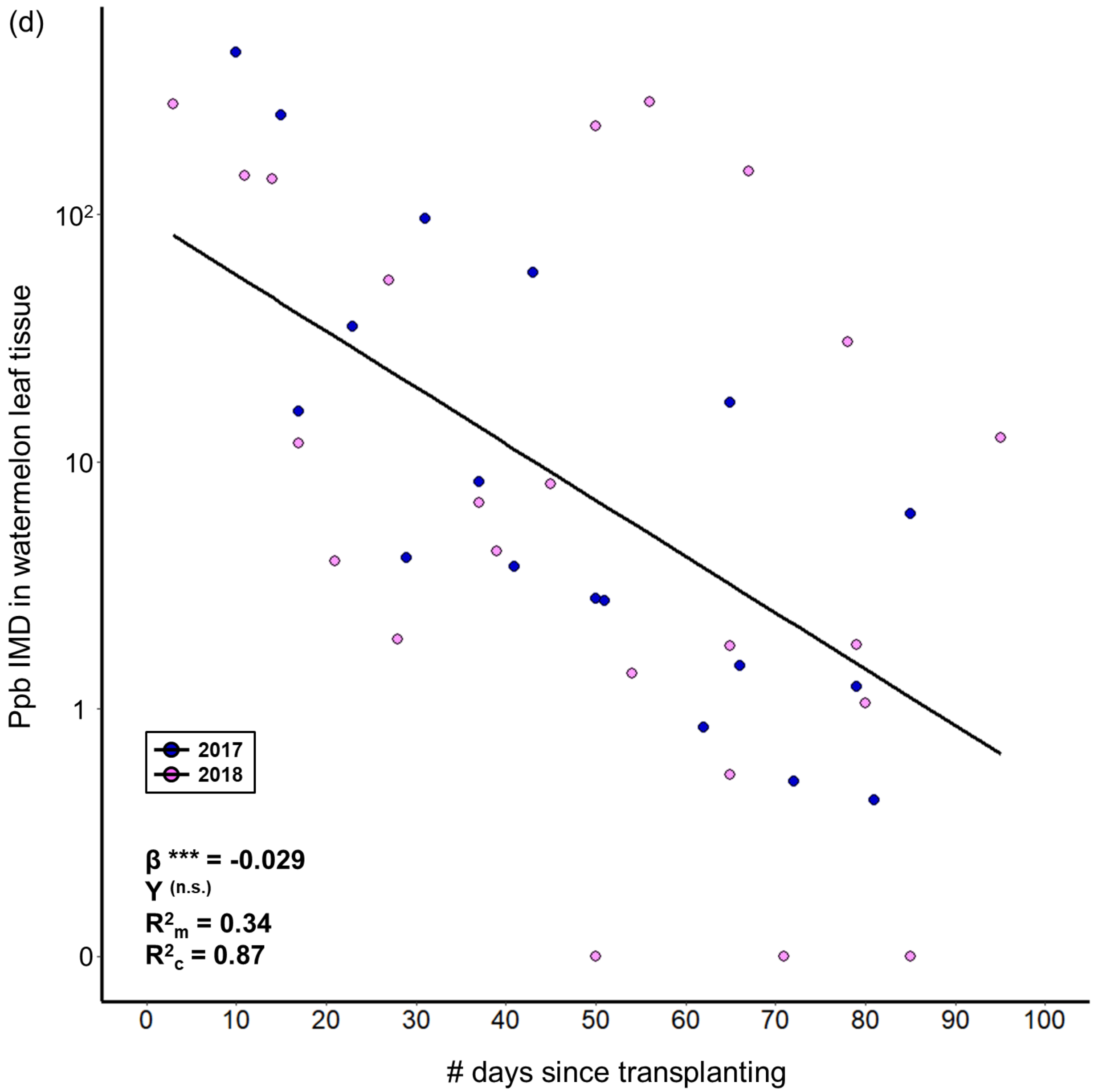

Figure S3d. Enlarged version of Figure 1d in the main text.

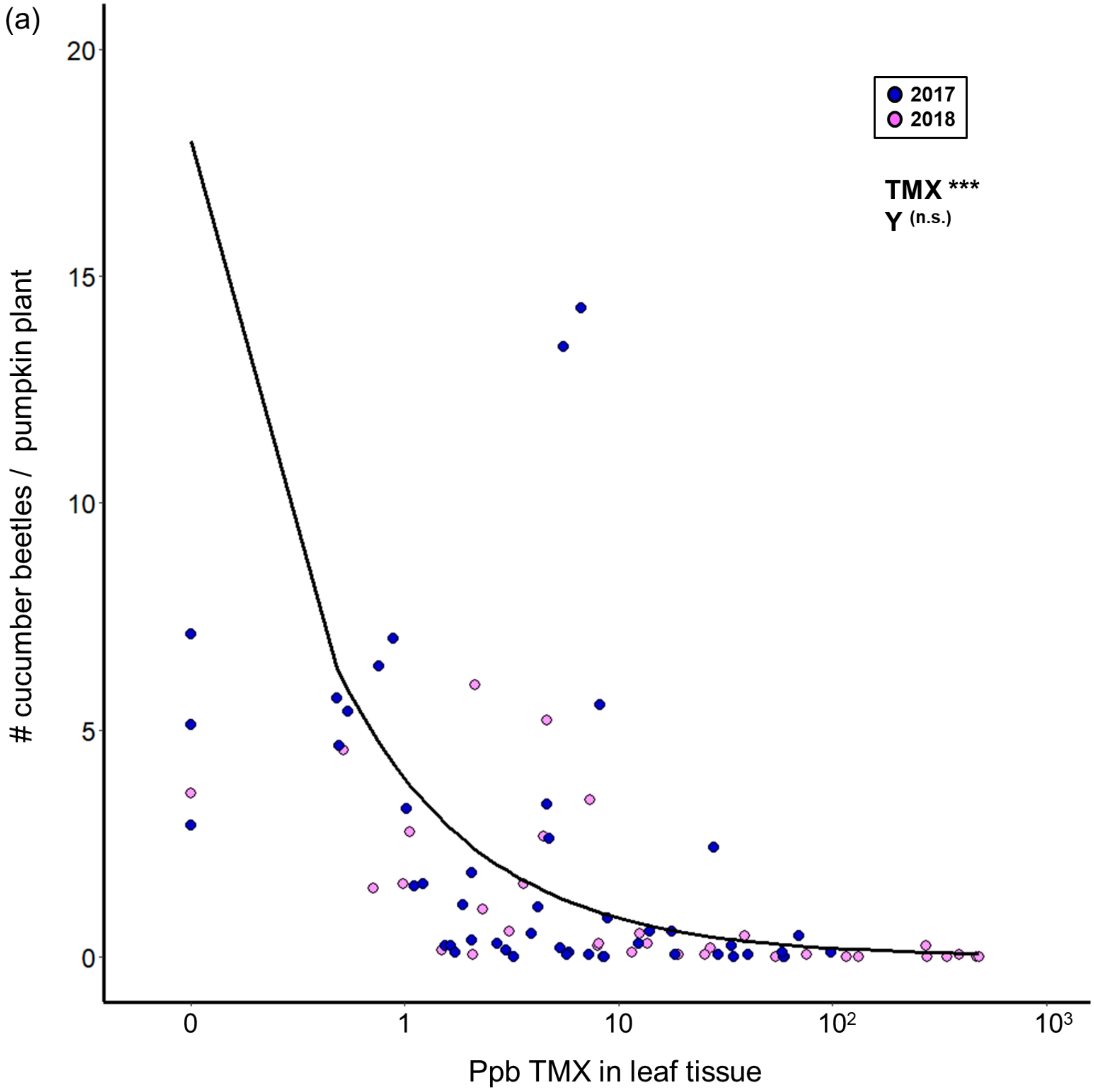

Figure S4a. Enlarged version of Figure 2a in the main text.

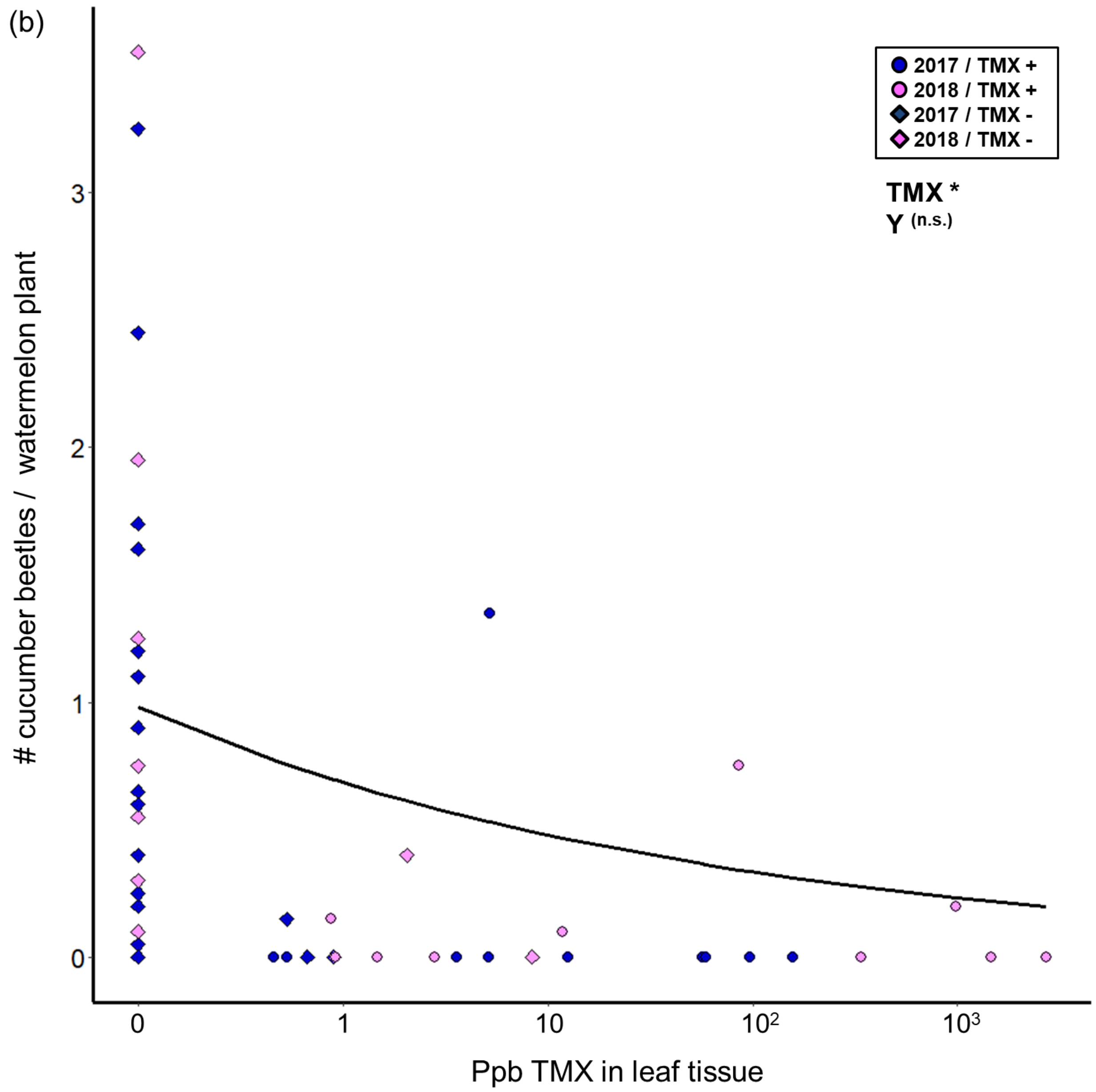

Figure S4b. Enlarged version of Figure 2b in the main text.

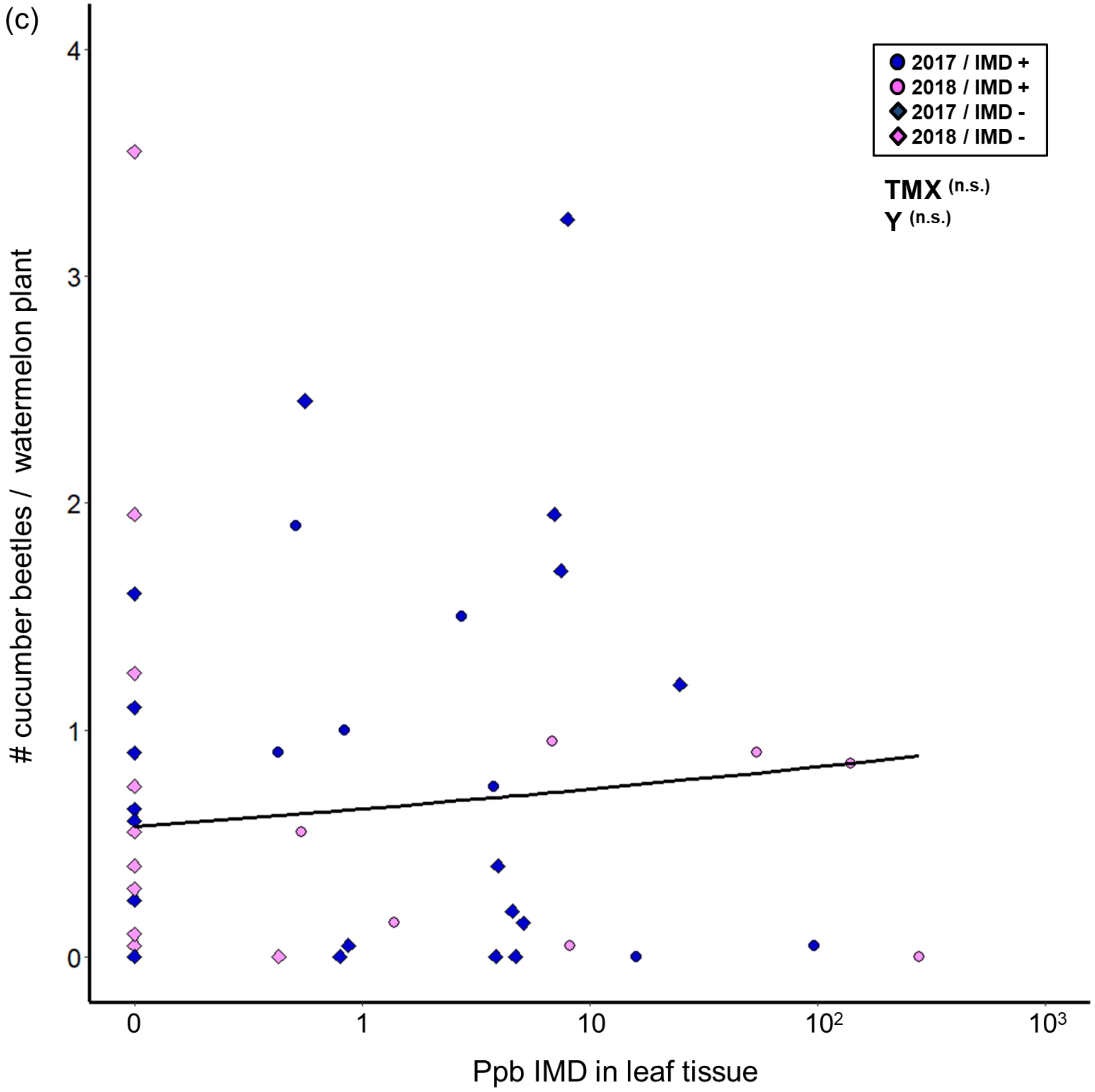

Figure S4c. Enlarged version of Figure 2c in the main text.

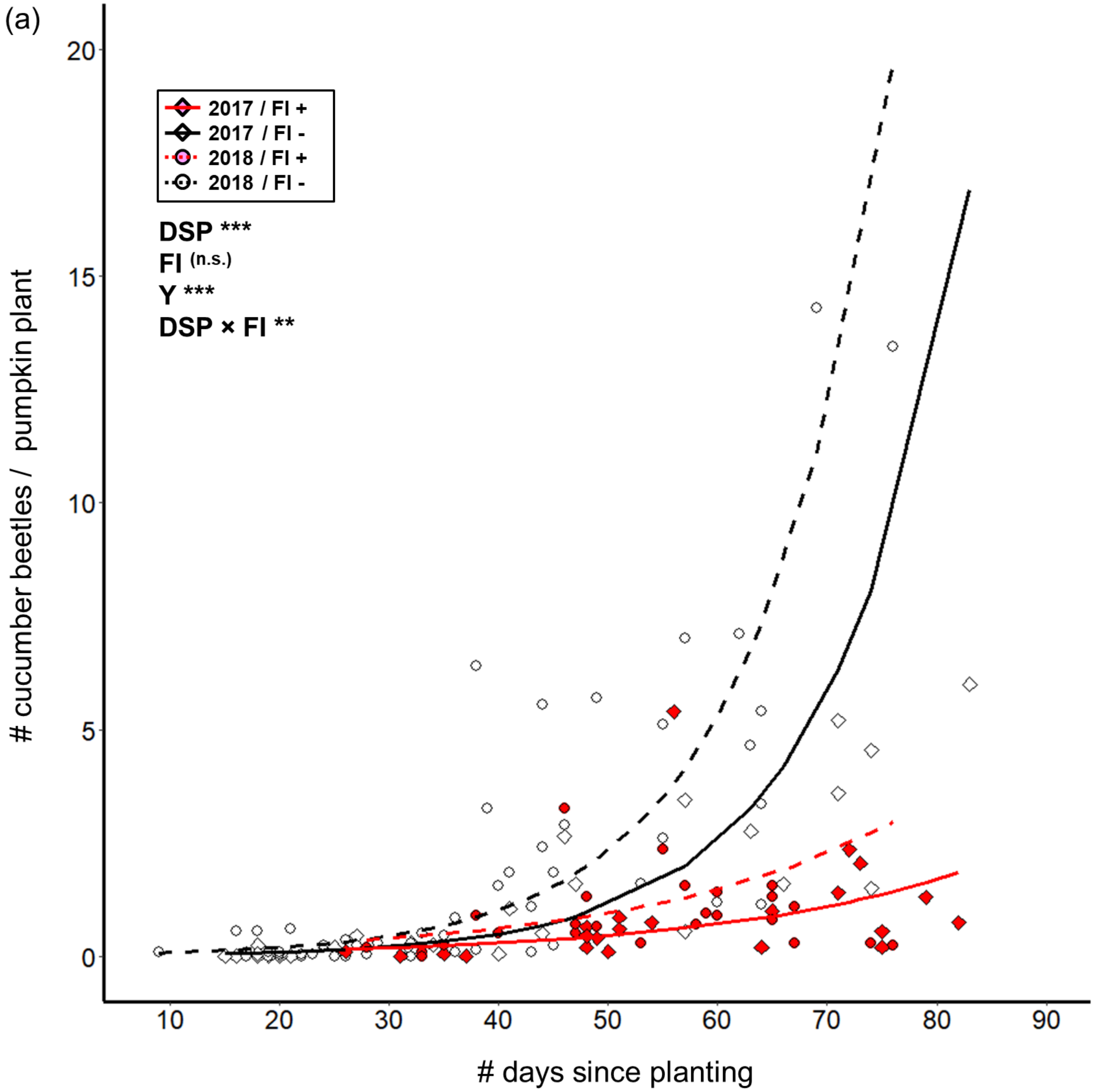

Figure S5a. Enlarged version of Figure 3a in the main text.

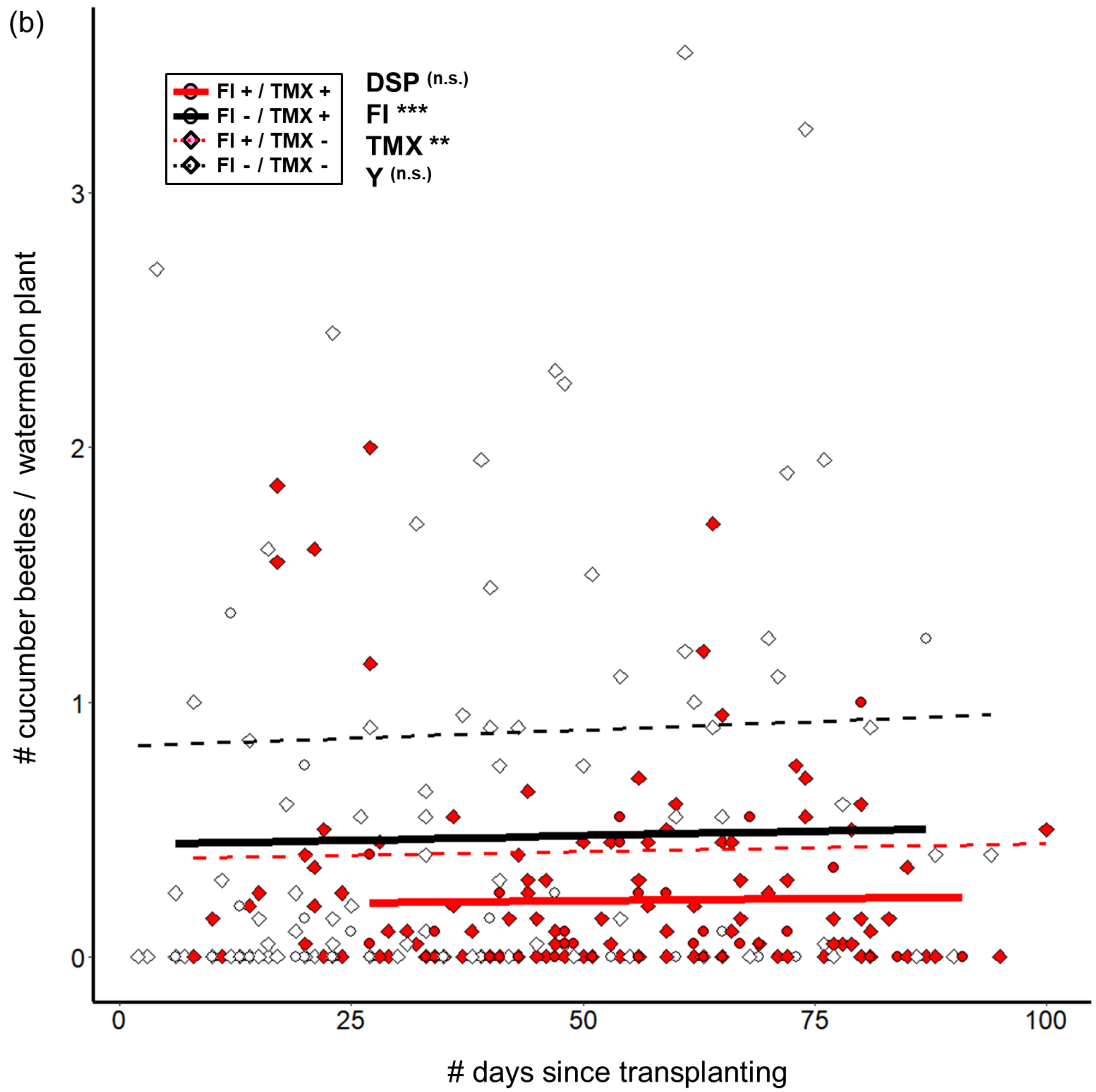

Figure S5b. Enlarged version of Figure 3b in the main text.

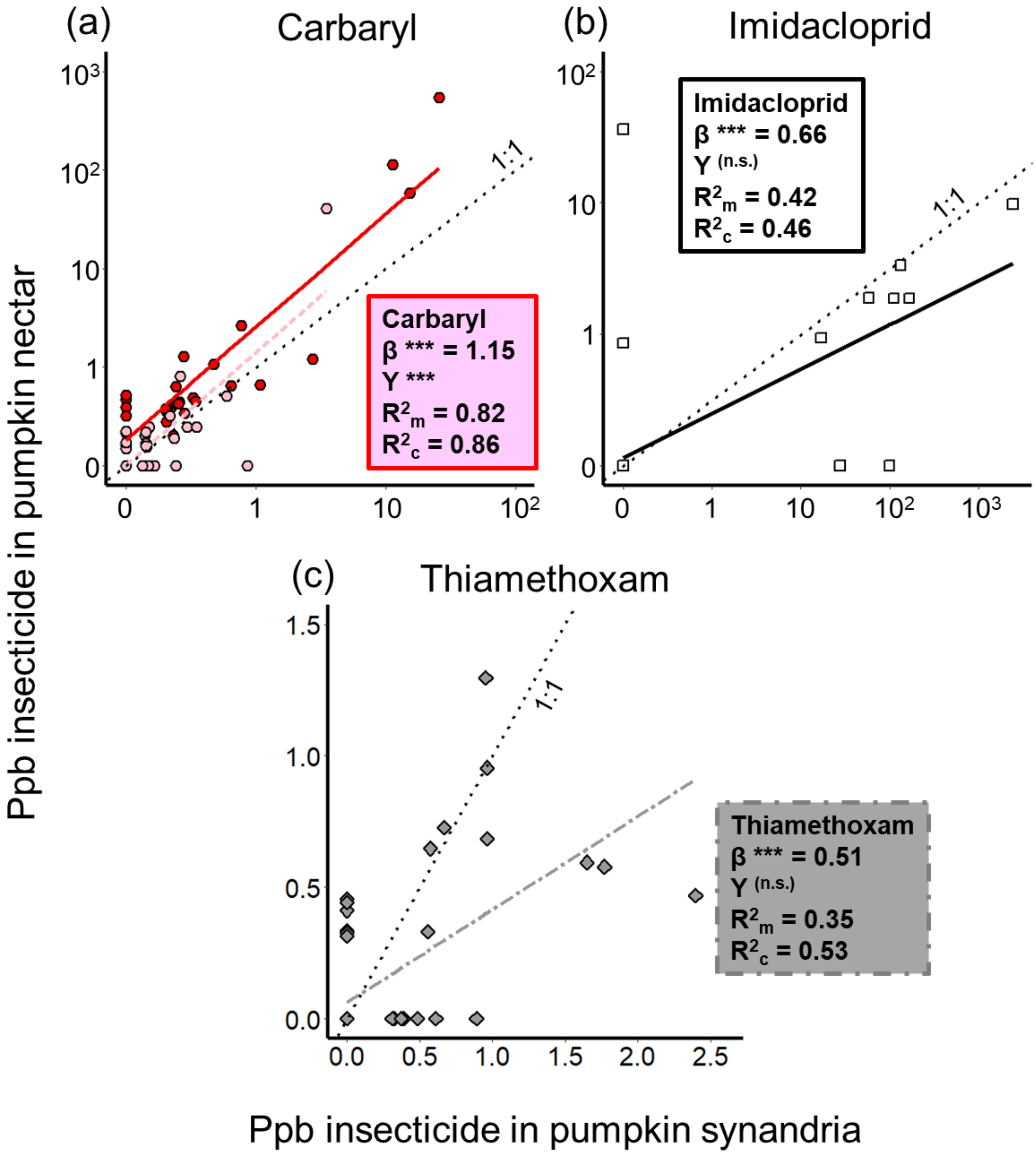

Figure S6. Data from Figure 5 of the main text, with each insecticide graphed individually. Note that panels (a) and (b) are graphed on a $\log_{10}$ scale, whereas panel (c) is graphed on a linear scale.

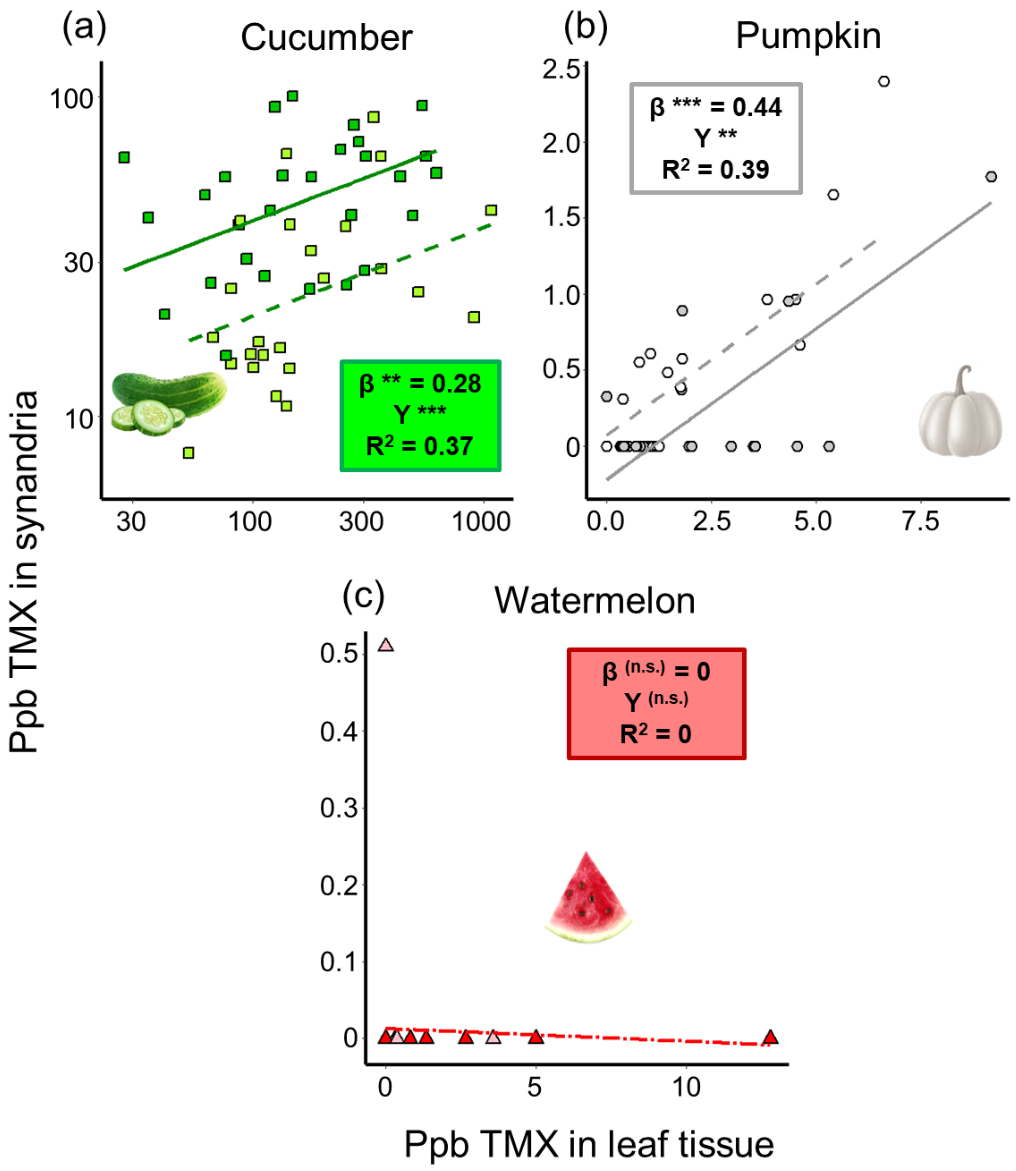

Figure S7. Data from Figure 6a of the main text, with each cropping system graphed individually. Note that panel (a) is graphed on a $\log_{10}$ scale, whereas panels (b) and (c) are graphed on a linear scale.

Table S1. Number of farm-year combinations involved in each analysis.

Figure	System	Year	No. farm-year combinations
1a	Cucumber: TMX	2017	16
1a	Cucumber: TMX	2018	14
1b	Pumpkin: TMX	2017	14
1b	Pumpkin: TMX	2018	16
1c	Watermelon: TMX	2017	4
1c	Watermelon: TMX	2018	3
1d	Watermelon: IMD	2017	4
1d	Watermelon: IMD	2018	3
2a	Pumpkin: TMX	2017	13
2a	Pumpkin: TMX	2018	15
2b	Watermelon: TMX	2017	8
2b	Watermelon: TMX	2018	9
2c	Watermelon: IMD	2017	7
2c	Watermelon: IMD	2018	8
3a	Pumpkin: FI +	2017	9
3a	Pumpkin: FI +	2018	9
3a	Pumpkin: FI-	2017	14
3a	Pumpkin: FI-	2018	16
3b	Watermelon: FI + / TMX +	2017	3
3b	Watermelon: FI + / TMX +	2018	2
3b	Watermelon: FI - / TMX +	2017	4
3b	Watermelon: FI - / TMX +	2018	3
3b	Watermelon: FI + / TMX -	2017	8
3b	Watermelon: FI + / TMX -	2018	8
3b	Watermelon: FI - / TMX -	2017	9
3b	Watermelon: FI - / TMX -	2018	10
4a-b	Cucumber: TMX	Both	30
4a-b	Pumpkin: TMX	Both	30
4a-b	Watermelon: TMX +	Both	7
4a-b	Watermelon: TMX -	Both	23
4a-b	Pumpkin: IMD +	Both	2
4a-b	Pumpkin: IMD -	Both	28
4a-b	Watermelon: IMD +	Both	8
4a-b	Watermelon: IMD -	Both	22
4a-b	Pumpkin: carbaryl +	Both	9
4a-b	Pumpkin: carbaryl -	Both	21

Table S1 (continued)

Figure	System	Year	No. farm-year combinations
5	Pumpkin: TMX	2017	14
5	Pumpkin: TMX	2018	16
5	Pumpkin: IMD	2017	14
5	Pumpkin: IMD	2018	16
5	Pumpkin: carbaryl	2017	14
5	Pumpkin: carbaryl	2018	16
6a	Cucumber: TMX	2017	16
6a	Cucumber: TMX	2018	14
6a	Pumpkin: TMX	2017	14
6a	Pumpkin: TMX	2018	16
6a	Watermelon: TMX	2017	14
6a	Watermelon: TMX	2018	14
6b	Watermelon: IMD	2017	14
6b	Watermelon: IMD	2018	14

Table S2. Post-hoc pairwise tests comparing cropping systems with respect to the slope of the relationship between leaf tissue neonicotinoid concentration and time since planting. Tests were performed using Tukey adjustment for multiple comparisons, and Kenward-Roger method for estimating degrees of freedom.

Contrast	*t* value	*P* value
Cucumber vs. pumpkin	3.87	0.0004
Cucumber vs. watermelon	7.03	< 0.0001
Pumpkin vs. watermelon	4.21	0.0001

Table S3. Post-hoc pairwise tests comparing pollen hazard quotients across combinations of cropping systems and insecticide. Tests were performed using Tukey adjustment for multiple comparisons, and Kenward-Roger method for estimating degrees of freedom.

Contrast	*t* value	*P* value
Cucumber TMX vs. pumpkin carbaryl	8.72	< 0.0001
Cucumber TMX vs. pumpkin IMD	2.19	0.25
Cucumber TMX vs. pumpkin TMX	15.6	< 0.0001
Pumpkin carbaryl vs. pumpkin IMD	2.66	0.090
Pumpkin carbaryl vs. pumpkin TMX	3.05	0.032
Pumpkin IMD vs. pumpkin TMX	4.44	0.0002
Watermelon IMD vs. cucumber TMX	8.05	< 0.0001
Watermelon IMD vs. pumpkin carbaryl	0.35	1.00
Watermelon IMD vs. pumpkin IMD	2.74	0.074
Watermelon IMD vs. pumpkin TMX	1.78	0.49
Watermelon IMD vs. watermelon TMX	1.47	0.68
Watermelon TMX vs. cucumber TMX	9.21	< 0.0001
Watermelon TMX vs. pumpkin carbaryl	1.9	0.41
Watermelon TMX vs. pumpkin IMD	3.78	0.003
Watermelon TMX vs. pumpkin TMX	0.15	1.00

Table S4. Post-hoc pairwise tests comparing insecticides with respect to the slope of the relationship between insecticide concentrations in pumpkin and nectar and synandrium tissue. Tests were performed using Tukey adjustment for multiple comparisons, and Kenward-Roger method for estimating degrees of freedom.

Contrast	*t* value	*P* value
Carbaryl vs. imidacloprid	4.25	0.0001
Carbaryl vs. thiamethoxam	4.68	< 0.0001
Imidacloprid vs. thiamethoxam	1.01	0.57

Table S5. Post-hoc pairwise tests comparing cropping systems with respect to the slope of the relationship between thiamethoxam concentrations in synandrium and leaf tissue. Tests were performed using Tukey adjustment for multiple comparisons, and Kenward-Roger method for estimating degrees of freedom.

Contrast	*t* value	*P* value
Cucumber vs. pumpkin	1.01	0.57
Watermelon vs. cucumber	4.25	0.0001
Watermelon vs. pumpkin	4.68	< 0.0001

Table S6. Output details of models associated with Figure 1.

Model	Variable	Estimate	Standard error	*t* value	*P* value
Fig. 1a (cucumber, thiamethoxam)	Intercept (2017)	4.25	0.08	50.13	< 0.001
Fig. 1a (cucumber, thiamethoxam)	Slope (days elapsed)	-0.05	0.00	-23.27	< 0.001
Fig. 1a (cucumber, thiamethoxam)	Year (2018)	-0.13	0.07	-1.85	0.068
Fig. 1b (pumpkin, thiamethoxam)	Intercept (2017)	2.55	0.16	16.35	< 0.001
Fig. 1b (pumpkin, thiamethoxam)	Slope (days elapsed)	-0.04	0.00	-18.55	< 0.001
Fig. 1b (pumpkin, thiamethoxam)	Year (2018)	-0.51	0.09	-5.98	< 0.001
Fig. 1c (watermelon, thiamethoxam)	Intercept (2017)	2.30	0.40	5.76	0.0014
Fig. 1c (watermelon, thiamethoxam)	Slope (days elapsed)	-0.02	0.00	-6.11	< 0.001
Fig. 1c (watermelon, thiamethoxam)	Year (2018)	-0.39	0.21	-1.83	0.075
Fig. 1d (watermelon, imidacloprid)	Intercept (2017)	2.40	0.45	5.35	0.0027
Fig. 1d (watermelon, imidacloprid)	Slope (days elapsed)	-0.03	0.00	-9.35	< 0.001
Fig. 1d (watermelon, imidacloprid)	Year (2018)	0.27	0.18	1.47	0.15

Table S7. Output details of models associated with Figure 2.

Model	Variable	Estimate	Standard error	*t* value	*P* value
Fig. 2a (pumpkin, thiamethoxam)	Intercept (2017)	3.86	0.21	18.60	< 0.001
Fig. 2a (pumpkin, thiamethoxam)	Slope (log10 ppb thiamethoxam)	-1.81	0.15	-12.42	< 0.001
Fig. 2a (pumpkin, thiamethoxam)	Year (2018)	-0.18	0.22	-0.82	0.42
Fig. 2a (pumpkin, thiamethoxam)	Sigma coefficient (log link)	-0.42	0.24	-1.74	0.086
Fig. 2a (pumpkin, thiamethoxam)	Nu coefficient (logit link)	-4.55	2.89	-1.57	0.12
Fig. 2b (watermelon, thiamethoxam)	Intercept (2017)	2.33	0.27	8.61	< 0.001
Fig. 2b (watermelon, thiamethoxam)	Slope (log10 ppb thiamethoxam)	-0.40	0.20	-1.99	0.051
Fig. 2b (watermelon, thiamethoxam)	Year (2018)	0.20	0.37	0.53	0.60
Fig. 2b (watermelon, thiamethoxam)	Sigma coefficient (log link)	0.02	0.39	0.04	0.97
Fig. 2b (watermelon, thiamethoxam)	Nu coefficient (logit link)	-0.31	0.33	-0.95	0.35
Fig. 2c (watermelon, imidacloprid)	Intercept (2017)	1.76	0.23	7.55	< 0.001
Fig. 2c (watermelon, imidacloprid)	Slope (log10 ppb imidacloprid)	-0.06	0.13	-0.42	0.68
Fig. 2c (watermelon, imidacloprid)	Year (2018)	0.20	0.27	0.76	0.45
Fig. 2c (watermelon, imidacloprid)	Sigma coefficient (log link)	2.79	0.27	10.26	< 0.001

Table S8. Output details of models associated with Figure 3.

Model	Variable	Estimate	Standard error	*t* value	*P* value
Fig. 3a (pumpkin)	Intercept (2017)	-1.06	0.33	-3.15	0.002
Fig. 3a (pumpkin)	Slope (days elapsed)	0.08	0.01	10.57	< 0.001
Fig. 3a (pumpkin)	Foliar insecticide (applied)	1.24	0.76	1.63	0.11
Fig. 3a (pumpkin)	Year (2018)	0.73	0.17	4.38	< 0.001
Fig. 3a (pumpkin)	Days elapsed x foliar insecticide	-0.04	0.01	-2.63	0.0096
Fig. 3a (pumpkin)	Sigma coefficient (log link)	-0.25	0.13	-1.89	0.060
Fig. 3a (pumpkin)	Nu coefficient (logit link)	-36.04	8638.68	0.00	1.00
Fig. 3b (watermelon)	Intercept (2017, no foliar / systemic insecticides)	2.59	0.26	9.86	< 0.001
Fig. 3b (watermelon)	Slope (days elapsed)	0.00	0.00	1.02	0.31
Fig. 3b (watermelon)	Foliar insecticide (applied)	-0.76	0.20	-3.77	0.0002
Fig. 3b (watermelon)	Year (2018)	-0.22	0.21	-1.05	0.29
Fig. 3b (watermelon)	Thiamethoxam (applied at transplanting)	-0.43	0.21	-2.09	0.038
Fig. 3b (watermelon)	Sigma coefficient (log link)	0.33	0.24	1.37	0.17
Fig. 3b (watermelon)	Nu coefficient (logit link)	-0.66	0.26	-2.54	0.012

Table S9. Output details of models associated with Figure 5.

Model	Variable	Estimate	Standard error	*t* value	*P* value
Fig. 5 (carbaryl)	Intercept (2017)	0.41	0.07	5.55	< 0.001
Fig. 5 (carbaryl)	Slope (log10 ppb carbaryl in synandria)	1.15	0.08	13.67	< 0.001
Fig. 5 (carbaryl)	Year (2018)	-0.26	0.08	-3.13	0.0029
Fig. 5 (imidacloprid)	Intercept (2017)	-0.26	0.11	-2.30	0.025
Fig. 5 (imidacloprid)	Slope (log10 ppb imidacloprid in synandria)	0.66	0.10	6.42	< 0.001
Fig. 5 (imidacloprid)	Year (2018)	-0.03	0.10	-0.27	0.7851
Fig. 5 (thiamethoxam)	Intercept (2017)	-0.37	0.10	-3.86	< 0.001
Fig. 5 (thiamethoxam)	Slope (log10 ppb thiamethoxam in synandria)	0.51	0.09	5.78	< 0.001
Fig. 5 (thiamethoxam)	Year (2018)	-0.08	0.07	-1.15	0.26

Table S10. Output details of models associated with Figure 6.

Model	Variable	Estimate	Standard error	*t* value	*P* value
Fig. 6a (cucumber, thiamethoxam)	Intercept (2017)	603.72	124.77	4.84	< 0.001
Fig. 6a (cucumber, thiamethoxam)	Slope (log10 ppb thiamethoxam in leaf tissue)	0.28	0.09	3.19	0.0025
Fig. 6a (cucumber, thiamethoxam)	Year (2018)	-0.30	0.06	-4.83	< 0.001
Fig. 6a (pumpkin, thiamethoxam)	Intercept (2017)	-621.37	198.74	-3.13	0.003
Fig. 6a (pumpkin, thiamethoxam)	Slope (log10 ppb thiamethoxam in leaf tissue)	0.44	0.08	5.62	< 0.001
Fig. 6a (pumpkin, thiamethoxam)	Year (2018)	0.31	0.10	3.12	0.003
Fig. 6a (watermelon, thiamethoxam)	Intercept (2017)	-76.91	75.60	-1.02	0.32
Fig. 6a (watermelon, thiamethoxam)	Slope (log 10 ppb thiamethoxam in leaf tissue)	-0.01	0.03	-0.27	0.79
Fig. 6a (watermelon, thiamethoxam)	Year (2018)	0.04	0.04	1.00	0.32
Fig. 6b (watermelon, imidacloprid)	Intercept (2017)	83.77	143.35	0.58	0.56
Fig. 6b (watermelon, imidacloprid)	Slope (log 10 ppb thiamethoxam in leaf tissue)	0.51	0.05	10.22	< 0.001
Fig. 6b (watermelon, imidacloprid)	Year (2018)	-0.04	0.07	-0.59	0.56
